# Deep-Learning-Assisted Underwater 3D Tactile Tensegrity

**DOI:** 10.34133/research.0062

**Published:** 2023-02-27

**Authors:** Peng Xu, Jiaxi Zheng, Jianhua Liu, Xiangyu Liu, Xinyu Wang, Siyuan Wang, Tangzhen Guan, Xianping Fu, Minyi Xu, Guangming Xie, Zhong Lin Wang

**Affiliations:** ^1^ Dalian Key Laboratory of Marine Micro/Nano Energy and Self-powered Systems, Marine Engineering College, Dalian Maritime University, Dalian 116026, China.; ^2^ School of Information Science and Technology, Dalian Maritime University, Dalian 116026, China.; ^3^ Intelligent Biomimetic Design Lab, College of Engineering, Peking University, Beijing 100871, China.; ^4^ Beijing Institute of Nanoenergy and Nanosystems, Chinese Academy of Sciences, Beijing 100871, China.; ^5^ School of Materials Science and Engineering, Georgia Institute of Technology, Atlanta, GA 30332-0245, USA.

## Abstract

The growth of underwater robotic applications in ocean exploration and research has created an urgent need for effective tactile sensing. Here, we propose an underwater 3-dimensional tactile tensegrity (U3DTT) based on soft self-powered triboelectric nanogenerators and deep-learning-assisted data analytics. This device can measure and distinguish the magnitude, location, and orientation of perturbations in real time from both flow field and interaction with obstacles and provide collision protection for underwater vehicles operation. It is enabled by the structure that mimics terrestrial animals’ musculoskeletal systems composed of both stiff bones and stretchable muscles. Moreover, when successfully integrated with underwater vehicles, the U3DTT shows advantages of multiple degrees of freedom in its shape modes, an ultrahigh sensitivity, and fast response times with a low cost and conformability. The real-time 3-dimensional pose of the U3DTT has been predicted with an average root-mean-square error of 0.76 in a water pool, indicating that this developed U3DTT is a promising technology in vehicles with tactile feedback.

## Introduction

As ocean exploration technology has advanced, autonomous underwater vehicles (AUVs) have become indispensable. A major mission of AUVs is to explore an unknown underwater environment with obstacles or places that are hard to visually observe [1–4]. Diverse sensor architectures have been proposed to aid AUVs in autonomously completing their missions, with collision/obstacle avoidance in a variety of environments [5,6]. Namely, with the aid of device data, AUVs possess the ability to foresee, recognize, and handle unexpected changes and then adjust if these competences do not satisfy a certain level [7–9]. However, when optical or sonic reflection problems occur in a narrow space, it remains challenging for AUVs to identify unknown underwater environments.

Tensegrity structures are typically volumetric mechanical structures composed of a set of separated rigid elements connected by a continuous network of tensional elements, mimicking terrestrial animals’ musculoskeletal systems composed of both stiff bones and stretchable muscles [10–13]. The Intelligent Robotics Group at the NASA Ames Research Center has been developing the SUPERball (Spherical Underactuated Planetary Exploration Robot) using tensegrity structures, which could provide a technological solution for planetary landing and exploration missions [14–16]. These light, low-cost robots make certain missions more practical, since their structural designs allow the system to passively adapt to external forces and redistribute loads effectively through a tension network [17,18]. On the basis of these characteristics, the tensegrity structure that can change its shape in response to an external force is capable of performing scientific measurements in narrow spaces. This capacity enables it to explore and recognize unknown underwater environments via its posture estimation without being dependent of the underwater optical or sonic devices. In addition, the structure can also provide high impact resilience with a low weight, making it a potential candidate for collision-resilient AUVs. It was interestingly found that tensegrity can be viewed as excellent tactile perception scheme for underwater vehicles exploring an unknown underwater environment [19]. However, little attention has been paid to tactile sensors based on tensegrity structures for use in the underwater 3-dimensional (3D) tactile perception.

In general, by employing tactile sensing, including the sensing of forces, force directions, the contact location, and the contact surface, as feedback parameters in a closed-loop control system, robots can excellently perform most manipulation tasks or explore unknown environments [20–24]. On this basis, several research groups have developed a kind of tactile sensor combined with resistive (contact-resistance-based) [25], piezoresistive [26,27], magnetic [28,29], or capacitive sensor arrays [30], resulting in sensing improvements of the sensitivity, response time, and linearity. Specifically, Boutry et al. [31] designed a biomimetic soft electronic tactile sensor composed of capacitive sensor arrays. The sensor has several advantages, including an improved sensitivity, minimal hysteresis, excellent cycling stability, and response times in the millisecond range. On the basis of a structure of whisker follicles, Beem et al. [32,33] designed an underwater tactile sensor using a piezoelectric sensor array for underwater motion perception. These devices typically require complex fabrication techniques and have limited degrees of freedom (DOFs) in their shape modes, limiting their general application in robot–environment interaction, particularly in underwater environments. Thus, it is still challenging to construct a 3D tactile sensor with ultrasensitivity, high deformability, and high scalability.

To date, triboelectric nanogenerators (TENGs) have not been developed into tactile sensors based on tensegrity structures for making scientific measurements in narrow places. Noticeably, the outputs of TENG-based sensors in response to deformation are characterized by low hysteresis and high repeatability, which simplifies the design of signal processing circuits [34–37]. Some triboelectric devices have been designed for irregular and ultralow-frequency energy harvesting [38–42], rotational and linear motion sensing [43], nanometer-scale motion monitoring [44], and external stimulus detection [45–47]. On the basis of these results, a TENG-based underwater 3D tactile tensegrity (U3DTT) with multiple DOFs may provide a simple scheme for robot–environment interactions, relying on passive tactile perception.

**Fig. 1.**
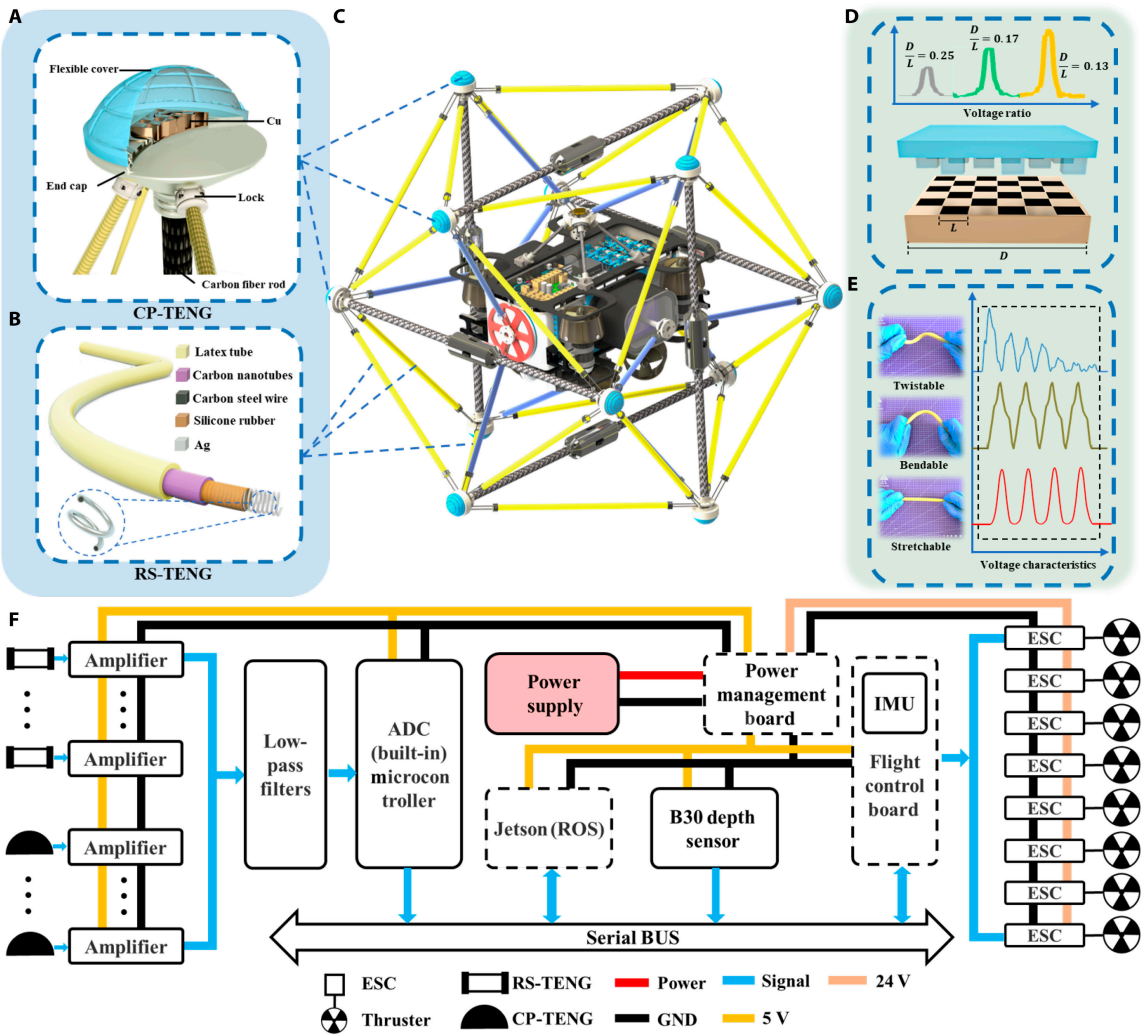
Construction diagram and applications of TENG based tensegrity . (A and B) Basic structures of the CP-TENG sensor (A) and RS-TENG sensor (B). (C) Structural diagram of tensegrity. (D) Voltage ratio of CP-TENG for different *D*/*L* values. (E) Characteristics of voltage curves of RS-TENG in different states: twistable, bendable, and stretchable states. (F) System-level block schematic of the triboelectric-tensegrity-based tactile perception system for AUVs, showing analog signal acquisition, processing transmission, electronic speed control (ESC), and thruster driven from the sensor array to AUV control. ADC, analog-to-digital converter; GND, ground.

In this work, a deep learning (DL)-enabled U3DTT based on a triboelectric mechanism was developed to construct an intelligent, self-powered, multi-DOF, and highly conformable 3D tactile perception system and provide collision resilience for underwater vehicles. The developed sensing system was built by integrating the voltage signals of triboelectric cap-shaped pressing (CP) sensors and rope-shaped stretchable (RS) sensors with advanced DL-based data analytics, as shown in Fig. 1. The CP-TENG sensor array was able to determine the location of the impact and the magnitude of the impact force. The RS-TENG sensor generated signals for measuring the tension network via contact separation between the electropositive carbon nanotubes (CNTs) and the electronegative silicone rubber. Moreover, assisted by integrated DL-based data analytics, 3D pose information of the U3DTT could be extracted from the output voltage of triboelectric sensors. Benefiting from the 1D signal of 2 types of sensors, huge computing resources can be saved compared to the conventional image and massive channel-based process, enabling faster data analytics for real-time applications in measuring both the surrounding flow field and interaction with obstacles.

## Results

### Basic structure and working mechanism of the U3DTT

The goal of the U3DTT is to provide an artificial 3D tactile feedback for collision-resilient AUVs to guide their operation and ensure them survive collisions at high speed. The deformation of the tensegrity structure should be small, so that the working thruster cannot affect the triboelectric device’s signal output in response to impact forces. In addition, the tensegrity structure should also be lightweight to ensure that its additional mass has limited influence on the AUV operation.

The U3DTT consisted mainly of a CP-TENG sensor installed at the end of a rigid carbon fiber rod and an RS-TENG sensor as the tensional cable used to connect 2 rods. As shown in Fig. 1A, a checkerboard-shaped block with grooves made of Cu was employed as the positive triboelectric component in the CP-TENG sensor, while a silicon rubber cover with the spinous structure located on the grooves was employed as the negative triboelectric layer due to its excellent toughness, stretchability, and ability to gain electrons. Moreover, the CP-TENG sensor can convert the flexible cover deformation into electrical signals under an external pressing force. When the sensor removed an external pressing force, the cover recovered its original state under the push-back force from the soft materials. As illustrated schematically in Fig. 1B, the structure of the RS-TENG sensor was mainly composed of silicone rubber chosen as the negative triboelectric component and coated evenly on the coiled wire (Ag-plated carbon steel), and CNTs were chosen as the positive triboelectric layer. The main body of the RS-TENG was sealed with a latex tube and a clamp in the presence of an air layer between the latex tube and the CNTs, ensuring that triboelectric signals could be generated under a stretching force in an underwater environment. The detailed fabrication of the RS-TENG sensor and the CP-TENG sensor is provided in Methods.

Leveraging DL technology, the U3DTT integrated with an AUV was successfully demonstrated to estimate its pose and perceive poorly modeled natural environments and provide a high impact resilience for the AUVs, as shown in Fig. 1C and Fig. S1. The tensegrity structure was designed to mimic terrestrial animals’ musculoskeletal systems, and it was used to construct a prototype of a light 3D tactile sensor with multiple DOFs in response to an external load. The signal readout strategies (Fig. 1D and E) include a voltage ratio caused by variations of the CP-TENG sensor structures and voltage characteristics in different states of the RS-TENG sensor, such as twistable, bendable, and stretchable states.

Figure 1F illustrates an overview of the process flow of the hardware and software, starting with an analog signal sampling, followed by conditioning, processing, transmission to a robot operating system (ROS) embedded with the DL algorithm for robust pose estimation of the tensegrity, and, finally, thruster control. The signal-conditioning techniques for each sensor were utilized using an analog circuit, as shown in Fig. S2, whose major function was to amplify the sensing signal and eliminate background noise. This guaranteed that the analog output of the TENG sensor was capable of accurately expressing the tensegrity pose information and was suitable for further conversion to a digital format using an analog-to-digital converter. A microcontroller possessed the communication ability to calibrate the conditioned signals to an on-board transceiver and transmit them to the ROS via a serial bus. The ROS on the Jetson board was developed with a built-in DL algorithm (transformer model). The detailed process of the transformer network operation is provided in the Learning-based controller and smart underwater tactile perception section. The tensegrity pose served as an input for the thruster’s controllers by combining the B30 depth sensor and the inertial measurement unit on the flight control board. This scheme enabled the U3DTT installed on the AUVs to express more accurately and robustly.

### Mechanical and electrical property characterization of RS-TENG

**Fig. 2.**
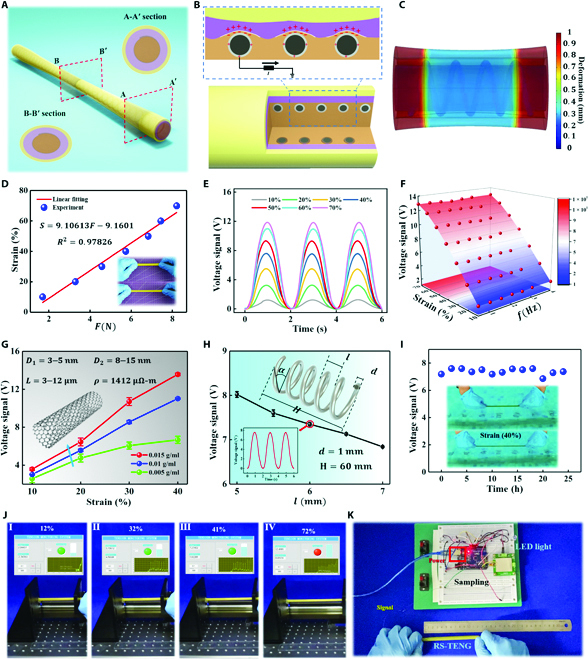
Working mechanism and characterization of the RS sensing unit. (A) Working mechanism of the RS-TENG sensor. Inset: Enlarged view of the sectional drawing. (B) Electrical potential distribution of the RS-TENG sensor. (C) Deformation simulation result of the RS-TENG sensor. (D) Stress–strain curve of the RS-TENG sensor. Inset: Photographs showing an initial state and the RS-TENG sensor. (E) Output voltage signals of the RS-TENG sensor with varying tensile strains at a stretching frequency of 0.5 Hz. (F) Relationship between the output signals, strains, and stretching frequencies. (G and H) Output voltage signals of the RS-TENG sensor with varying (G) CNT concentration (CNT parameters: inside diameter *D*_1_ = 3 to 5 nm, outer diameter *D*_2_ = 8 to 5 nm, length *L* = 3 to 12 μm, and resistivity *ρ* = 1, 412 μΩ-m) and (H) pitch of the steel wire. (I) Underwater stability of the RS-TENG sensor. (J) Schematic of the visualization interface and experimental setup. (K) Experiments with RS-TENG controlling LED lights.

The RS-TENG sensor was capable of actively converting stretching forces into distinguishable electrical signals, as shown in Fig. 2A, where a coiled wire made of Ag-plated carbon steel was chosen as the electrode. This design of the coiled structure could improve the stretchability of the electrode and ensure that the RS-TENG sensor maintained a decent electrical output, even under extreme stretching. The insets of Fig. 2A show 2 enlarged shapes of the sectional drawing located at different positions of the RS-TENG sensor, in which, because of the difference of the electron affinity between the silicone rubber and CNTs, an electric potential was induced by varying the contact area between the 2 materials during the stretch–release cycles. The silicone rubber layer had a strong ability to attract electrons, while the CNT layer tended to lose them, resulting in the generation of triboelectric static charges when the 2 materials approached each other, as schematically shown in Fig. 2B. The triboelectric potential drove electrons to flow along an external circuit from the shielding wire to the ground, while the positive charges built up on the surface of the wire-coiled electrode due to the electrostatic induction effect. Figure S3 shows a more detailed illustration of the electron transfer process during a stretch–release cycle. To visualize the strain and electrical potential distributions between the 2 triboelectric layers, COMSOL Multiphysics was used to verify the working principle, as shown in Fig. 2C and Fig. S4.

To quantify the RS-TENG sensor’s mechanical and electrical characteristics, a linear motor equipped with a force sensor was used to simulate a cyclic stretching force, as shown in Fig. S5. Figure 2D shows that the strain (from 10% to 70%) of the RS-TENG sensor increased with the increase in the stretching force. It is worth noting that the strain of the tensional cables reached a maximum value of 30% when the U3DTT operated under a high load. Namely, the 70% strain design of the RS-TENG sensor was far beyond the requirement for U3DTT operation. Figure 2E shows the output signal at a stretching frequency of 0.5 Hz as the strains varied from 10% to 70%. Furthermore, the relationship between the voltage (*U*) and external stimuli (*ϵ*) (*U* = 0.18086*ϵ* − 0.15857) is presented in Fig. S6, where the correlation coefficient *R*^2^ = 0.98627 indicated that the external stimuli and the output voltage had an approximate linear relationship. It was proven that a reliable electrical output of the RS-TENG sensor could be directly applied to a practical device. The RS-TENG sensor sensitivity is denoted as *S*_1_ = Δ*U*/Δ*F*. We obtained an excellent sensitivity of 1.8 V/N over a wide strain range and response times of 17 ms (Fig. S7A). To visualize the influence of each factor on the output signal, Fig. 2F depicts the relationship between the output voltage, strain, and stretching frequency, where the slope of the equal-height line reflected the strength of the effects of the 2 factors. As the stretching frequency increased, the output voltage remained almost unchanged. Figure S7B also shows the influence of the stretching frequency from 0.5 to 4 Hz on the voltage signal, which stayed around 11.84 V under a strain of 70%.

Moreover, we measured the influence of the silicone rubber–CNT composite with different CNT concentrations on the output voltage, as shown in Fig. 2G. The output voltage increased with the increase in the CNT concentration. In particular, cross-sectional scanning electron microscopy images of the composites with different CNT concentrations are shown in Fig. S8. The comparison of Fig. S8A and B with Fig. S8C shows that the surface roughness of the composite increased gradually with the CNT concentration, which could result from the disordered mixing of CNTs to a large degree. In addition, the increase ratio of the output voltage was more significant in the strain range from 30% to 40%, guaranteeing an accurate reflection of the stretching force through the voltage signal. The structural parameter of the coiled wire is also a significant indicator for the extreme stretching strain in practical applications that require long-term usage. The Young’s modulus of silicone rubber (3.7895 × 10^6^ N/m^2^) was much smaller than that of the Ag-plated carbon steel wire (2 × 10^11^ N/m^2^), which caused the value of the extreme stretching strain to be mostly governed by the tensile characteristics of the coiled wire. From the inset in Fig. 2H, we obtained the relationship between the spiral angle *α* and the pitch *l* [*α* = tan^−1^(*l*/*πD*)]. Furthermore, the output voltages with different values of the pitch *l* of the coiled wire were acquired under a strain of 40%, as shown in Fig. 2H. The output signal slightly decreased with the elongation *l*, which was attributed to the change in the contact area between the 2 triboelectric materials. In addition, the RS-TENG sensor, as a robot-compatible device, should have long-time durability under strain when subjected to robot–environment interactions. As demonstrated in Fig. S9, it maintained a stable electrical output under a strain of 40% after the application of 1, 000 continuous stretching force cycles. It is worth noting that a 40% strain could satisfy the requirement for the U3DTT operating under an impact force. Figure 2I shows the output voltage of the RS-TENG sensor in the underwater environment after repeated impacts from a cyclic stretching force, where the inset to Fig. 2I shows that the 20-cm-long RS-TENG sensor could be stretched to 28 cm. On the basis of the comparison with the output voltage of the RS-TENG sensor in the air, the underwater environment nearly did not degrade the performance of the voltage amplitude under a strain of 40%, even after being soaked in an indoor pool involving long-time testing. It was proven that the air layer between the latex tube and CNTs ensured that triboelectric signals were generated under a stretching force without the interference of the underwater environment.

To illustrate the real-time monitoring ability of the RS-TENG sensor, a tension monitoring system was designed using the fitted relationship between the stretching force and the voltage, as shown in Fig. 2J (also see Movie S1). When the linear motor stretched the RS-TENG sensor, the real-time signals under the cyclic stretching force were directly observed on a Keithley (6514) electrometer. The signals of the cyclic stretching force were recorded under different strains, showing that the stretching force could be clearly recognized on the basis of the voltage magnitude. In conclusion, the RS-TENG sensor possesses a promising sensing ability that enables real-time monitoring of the continuous stretching motion by extracting the voltage magnitude. Furthermore, Fig. 2K shows an electronic setup for controlling light-emitting diodes (LEDs), wherein the LEDs were installed in a line and an Arduino Due R3 was used as a circuit board to perform signal sampling and data processing. Data processing consisted of 2 stages: system initialization and event detection by judging the levels of the peak voltage for the RS-TENG sensor. This framework provides a triggering capability for driving different quantities of LED lights. An electrical diagram is depicted in Fig. S10. In the demonstration, the quantity of the turned-on LED lights increased with the increase in the stretching force (see Movie S2). These results demonstrate the potential for the RS-TENG to recognize its strain, which means that RS-TENG can be integrated into the tensegrity structure and used to complete numerous perception tasks.

### Mechanical and electrical property characterization of CP-TENG

**Fig. 3.**
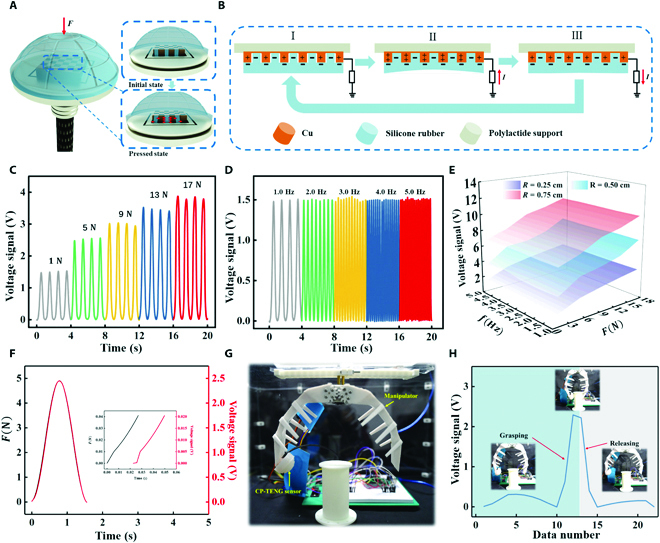
Working mechanism and characterization of the cap-shaped sensing unit. (A) Working mechanism of the CP-TENG sensor. Inset: Enlarged view of the pressing process. (B) Electrical potential distribution when pressing the CP-TENG sensor. (C to D) Output voltage signals when pressing the CP-TENG sensor with varying (C) force magnitudes at a pressing frequency of 1 Hz, (D) frequencies at a constant force of 1 N, (E) contact areas. (F) Response time measurement of the CP-TENG sensor. (G and H) Photographs of the CP-TENG sensor integrated into the sensing system to control a manipulator and the corresponding voltage signal.

Figure 3A and B shows the working mechanism of the CP-TENG sensor. When flexible cover deformation occurred under an external pressing force, due to triboelectrification, electrostatic induction, and Cu having a greater electronegativity than silicone rubber, the electrodes emitted electrons through an external circuit, thus generating a displacement current, as shown in Fig. 3B(II). When the pressing force was removed from the CP-TENG, the Cu electrode was separated from the silicone rubber. The charge distribution returned to its initial state, while the induced negative charges in the electrodes flowed back to the ground, as shown in Fig. 3B(III). This means that the whole generation cycle was complete. The COMSOL software was employed to simulate the potential distribution results between 2 triboelectric materials (see Fig. S11).

Figure 3C shows the generated voltage signals under a pressing frequency of 1 Hz as the force varied from 1 to 17 N. Furthermore, the relationship between the voltage and the external stimuli (*U* = 0.1395*F* + 1.6165, *R*^2^ = 0.97415) is presented in Fig. S12. The CP-TENG sensor sensitivity is denoted as *S*_2_ = Δ*U*/Δ*F*, which achieved a lower sensitivity of 0.15 V/N compared to that of *S*_1_. This means that the underwater disturbance had little effect on the voltage signal of the device. However, the device output increased significantly when the flexible cover was pressed by the heavy impact force from a collision with an unknown obstacle. The characteristic would allow the location estimation of heavy external forces and an antidisturbance sensing capability. Figure 3D describes the influence of the pressing frequency from 1 to 5 Hz on the voltage signal under a force of 1 N. As the pressing frequency increased, the output voltage remained almost unchanged (also see Fig. S13). In addition, the flexible cover served as a device for measuring the total contact area. As depicted in Fig. 3E, increasing the contact area resulted in a rise in the voltage signals.

We also evaluated the CP-TENG sensors’ response times, a critical indicator for evaluating the performance of the U3DTT for obtaining real-time tactile perception information. Figure 3F shows that the response times were <30 ms, proving the excellent synchronization between the voltage signal and the corresponding pressing force. To further demonstrate the real-time control capability of the CP-TENG sensor, a device integrated with a manipulator was prepared to perform a grasping task, as shown in Fig. 3G, where the voltage from the CP-TENG sensor as a feedback signal was provided with the grasping system (see Fig. S14). Once the peak voltage from the sensor reached the set value, the manipulator stopped the grasping action and then released the grasped object. Moreover, the real-time voltage signal is shown in Fig. 3H (see also Movie S3). The gripping and releasing motions of the manipulator can be recognized from the rising and falling edges of the voltage signal. Thus, the CP-TENG sensor can be employed for real-time perception.

### Learning-based controller and smart underwater tactile perception

**Fig. 4.**
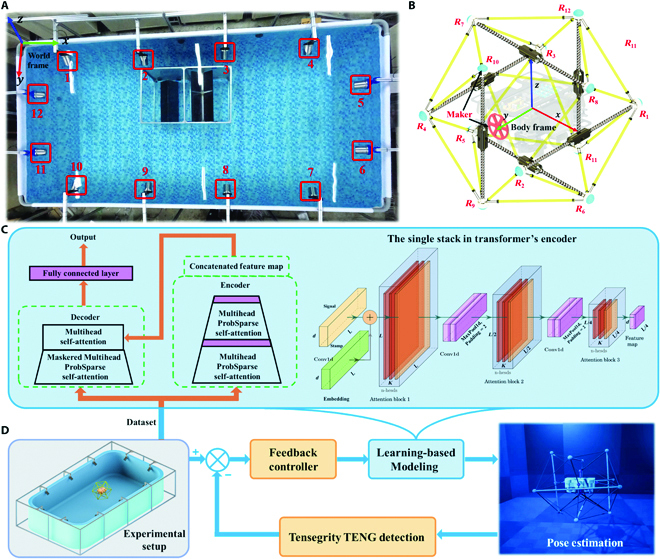
Components of the experimental setup. (A) Motion capture area. (B) Nodes of U3DTT and the considered reference frames. (C) Overview of the learning-based model. Twenty-four RS-TENG sensors signals, along with 12 CP-TENG sensors signal, acting as inputs of a transformer network, which mapped to the 12 node positions. (D) Feedback control loop. With this learned inverse mapping, the proposed tactile servoing controller allowed the AUV to autonomously explore the surrounding environment when optical or sonic reflection problems occur in a narrow space.

The experimental setup, including an indoor water pool (5.6 m × 2.7 m × 1.5 m), different shaped obstacles, and position-data acquisition by an optical motion capture system, is depicted in Fig. 4A and Fig. S15. A system with 12 motion capture cameras (DS-2XE8245FWD) was adopted for the measurement of the deformation of the tensegrity structure. The GSM7212 switch was used to link the synchronization device Optitrack eSync to the motion capture cameras. To enable the motion-capture system to obtain the node positions correctly, a hemispherical flexible cover and a circular metal plate were utilized as markers. For simplicity, the nodes are denoted as *R_i_* in Fig. 4B, where *i* = 1, 2, 3, …, 12. These nodes were connected by 6 rods and 24 tensional cables. We denote the connectivity of the nodes with functions *N_c_*, defined as follows:

$$N_{c}\left(R_{i},R_{j}\right)=\left\{\begin{array}{c} l,\text{if tensional cable}\;l\;\text{connects}\;R_{i}\;\text{and}\;R_{j}\\ 0 \end{array}\right..$$ (1)

For a single sample, the position data of the 12 moving nodes was obtained by the motion-capture system, while the voltage values of 24 RS-TENG sensors and 12 CP-TENG sensors were measured by the microcontroller via the analog-to-digital converter, which represented the variation in the intrinsic properties of the tensegrity structure.

A tactile servoing controller was implemented to explore the surrounding environment via estimating the real-time 3D pose of the U3DTT. The precondition was to develop an adaptive dynamic model that approximated the inverse mapping from the TENG sensor signals to the tensegrity pose. Conventional dynamic approaches demand an accurate description of both the tensegrity structural parameters and actuation response. It remains a well-known challenge for processing multisensor signals coupled with the extraction of relevant features, although a single TENG sensor had an approximate linear relationship between the external stimuli and the voltage. In comparison, data-driven techniques [48] such as DL approaches may take into account modeling uncertainties from the actual operation data, eliminating the requirement for hand-eye calibration. The given learning-based controller was initialized by training a transformer model using time-varying RS-TENG sensor signals and CP-TENG sensor signals as the input and the corresponding node position changes [*R_i_* = (*x_i_*, *y_i_*, *z_i_*)] as the output, as shown in Fig. 4C. The model was trained using relative positions for the nodes in the body frame rather than the world frame, which made it more robust to variations in the surrounding environment. We utilized a transformer model supplied by the PyTorch toolkit, where the self-attention mechanism was employed to make improvements to the network components and conduct extensive experiments. The transformer model contained a 3-layer stack and a one-layer stack (1/4 input) in the encoder, a 2-layer decoder, and an Adam optimizer with a learning rate of 0.0001. The detailed structural parameters of the transformer model are available in [49]. The network was trained using the Jetson NX edge computing platform (4/6 core 1,400 MHz) on the AUV. Moreover, to visualize the tensegrity structure pose monitoring in a no-camera underwater environment, pose estimation in real space could be real-time projected to a virtual space. The overall structure of the tactile servoing controller is shown in Fig. 4D.

### Underwater characterization of the U3DTT integrated with the AUV

**Fig. 5.**
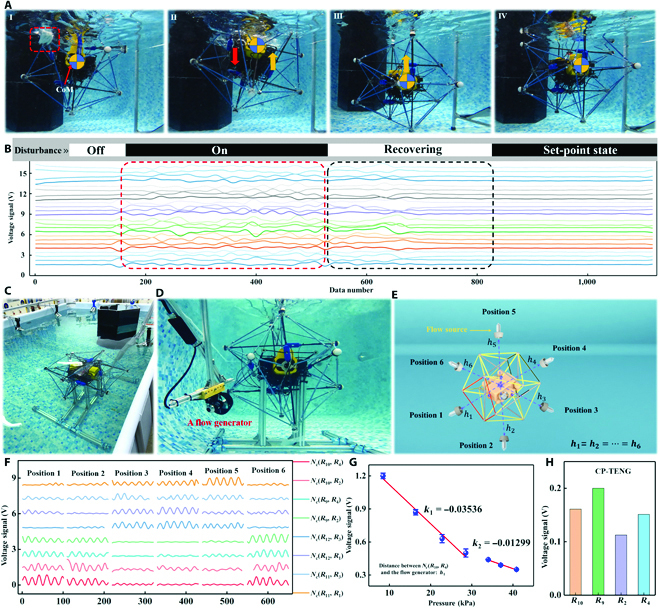
Experimental results with U3DTT integrated with the AUV. (A) AUV subjected to a disturbance automatically recovering the set-point state. (B) Voltage signal of U3DTT response to an impact force. (C and D) AUV with the U3DTT installed on the metal rack. (E) Position of the water flow generator around the U3DTT. (F and G) Voltage signal of RS-TENG response to (F) the flow field and (G) different prestresses. (H) Voltage signal of CP-TENG in response to the flow field.

An AUV subjected to external disturbances generally results in a chattering phenomenon in practical environments. This affects the performance of an RS-TENG sensor with excellent sensitivity, which makes it more difficult to analyze the pose of the U3DTT. For steady pose analysis of the U3DTT, the AUV’s antidisturbance control should be considered first in the underwater operation. Here, a structural design with 4 horizontal propellers and 4 vertical propellers was adopted, and there was no interference between the inflow and outflow of the vertical and horizontal propellers, thereby reducing the chattering phenomenon in practical applications. Moreover, a complementary filter was used to compensate for the drift, which affected the gyroscope measurements, and attenuate high-frequency signals, which affected the accelerometer-like vibrations. As a result, the proportional–integral–derivative controller was able to adaptively adjust the proportional–integral–derivative gain for an improved antidisturbance capacity. The robustness of the AUV’s antidisturbance control was subsequently tested, as shown in Fig. 5A. In the antidisturbance testing, a random impact force was applied to the AUV in a set-point state, mainly resulting in a longitudinal position change of the AUV’s center of mass, as shown in Fig. 5A(I and II) (also see Movie S4). The red dashed box in Fig. 5B highlights the voltage signal ripples of the U3DTT, which resulted in a chattering phenomenon of the AUV due to the shifted center of gravity and the unbalanced pitching moment on the vehicle caused by the impact force. When the impact force was removed from the U3DTT, due to the inertial forces during the dropping process of vehicles, the AUV with a U3DTT continued to dive down to the balance depth, as depicted in Fig. 5A(III). The black dotted box in Fig. 5B shows that the voltage gradually flattened. This was because with the aid of data from the inertial sensors and the complementary filter, the AUV was capable of rapidly recovering the set position and attitude without the chattering phenomenon, as shown in Fig. 5A(IV).

AUVs with the U3DTT typically conducted their mission autonomously in the background flow field. Thus, we consider the influence of the flow field on the output voltage. As shown in Fig. 5C and D, the AUV with the U3DTT was placed on a metal rack constructed of section steel. This configuration eliminated the influence of the AUV operation. A flow generator was positioned in several locations around the U3DTT, with a constant relative distance between them, as shown in Fig. 5E. Because of the symmetric structure of the U3DTT, the voltages of the sensors arranged on 4 open isosceles triangles (red lines and black lines) were analyzed to reduce the information complexity. The signal peak showed the influence of changing the position of the flow generator, as shown in Fig. 5F. For the RS-TENG sensors located at the 2 open isosceles triangles formed by the red lines, the signal peaks at positions 1, 2, and 6 were larger than those at positions 3, 4, and 5. The main reason was that bluff bodies (AUV) served to dampen the energy of the water flow, and the RS-TENG sensors were located at the 2 open isosceles triangles formed by the black lines, allowing them to avoid the vortex shedding from the bluff bodies. This characteristic is of great significance for distinguishing different flow directions. Since the stable equilibrium configuration for the U3DTT was achieved by the prestress of the tension network, there was a little difference in the voltage between *N_c_*(*R*_10_, *R*_4_) and *N_c_*(*R*_9_, *R*_2_). In particular, Fig. 5G shows that the voltage decreased slowly as the prestress increased. The prestress could reduce the influence of the working load from the flow field on the output signal. It is worth noting that the working sensitivity of the RS-TENG also decreased as with the increase in the prestress. In addition, Fig. 5H shows the signal peak of the CP-TENG (*R*_2_, *R*_4_, *R*_9_, *R*_10_) at position 1, in which the flow field had little influence of CP-TENG on the voltage due to its lower sensitivity.

**Fig. 6.**
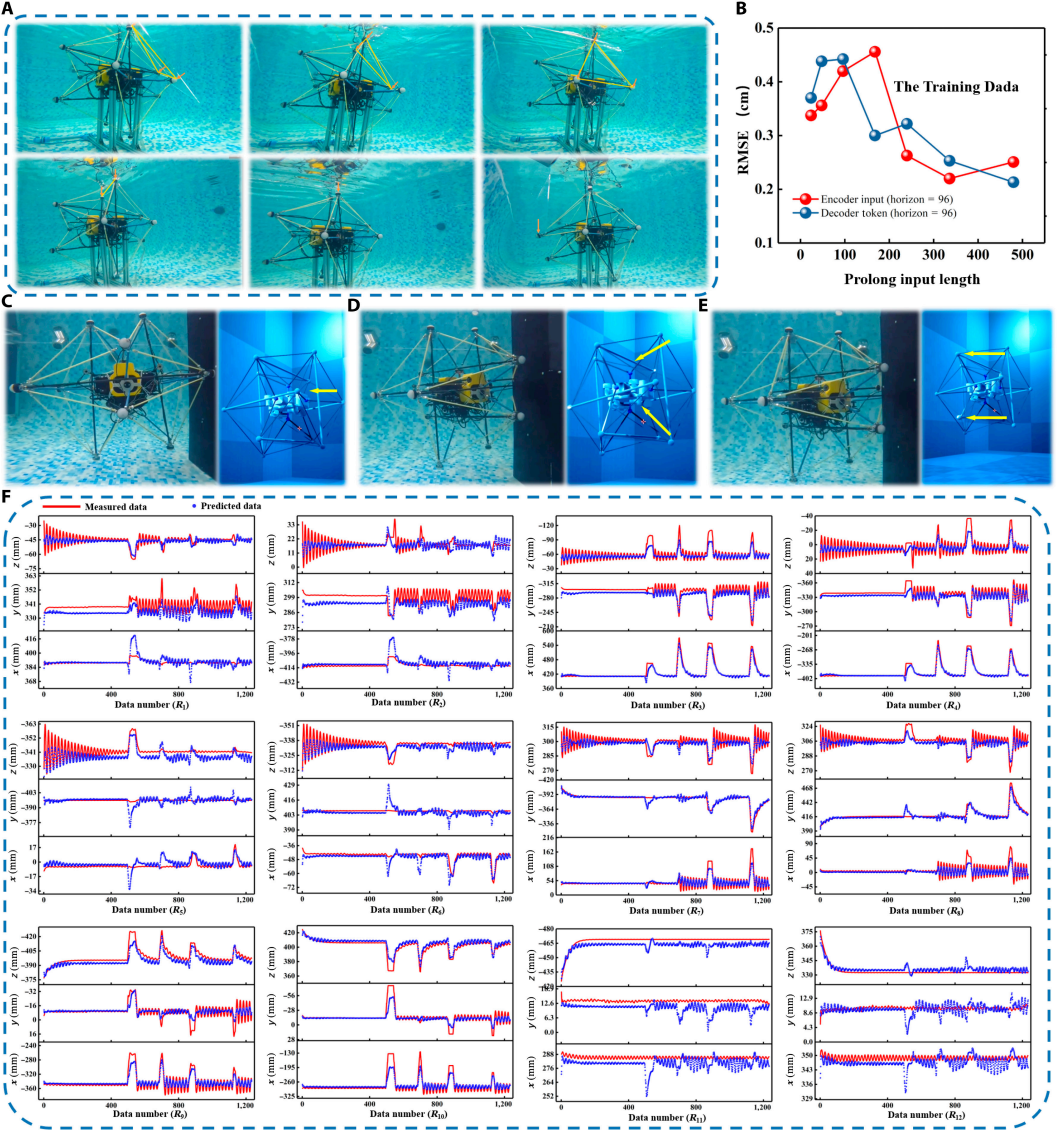
DL-assisted underwater tactile perception system. (A) Squeeze interaction experiments with the forcesensing tensegrity for collecting data. (B) Parameter sensitivity of input length. (C to E) Demonstration of the different position of collisions in real-time sensing. (F) Position predictions for 12 nodes (red line is actual data, and blue point is predicted data).

For gathering data from physical interactions between the tensegrity and its surroundings, the human operator used a transparent acrylic sheet to perform the compressing action toward isosceles triangle surfaces in different orientations, as shown in Fig. 6A (also see Movie S5). Timestamped data of the U3DTT were recorded on a microSD card at 120 Hz by the Jetson board. Fifty samples were collected and divided randomly into training/testing sets in the ratio of 80/20, respectively. The total number of epochs was set to 10 equipped with a suitable early stopping rule, in which we used the specified comparison techniques and a batch size of 32. From Table S1, the average root-mean-square error (RMSE) for the training data was around 0.3373, while the average RMSE for the testing data was about 1.732. In particular, the RMSE for case 41 was roughly 3.7, whereas it was 0.9 for case 47. When predicting sequences was set to 50, the initially prolonging input length reduced performance, but further increasing led the RMSE to drop because of the addition of repeating short-term patterns in Fig. 6B.

Moreover, to demonstrate the practical usage of the proposed device, the virtual environment was built to visualize the real-time tensegrity pose in the underwater through the DL prediction. Unlike the camera-based monitoring that normally involves video-taken concerns, this monitoring system employed a digital twin of the tensegrity structure to show the deformation of the U3DTT based on the interaction between the tensegrity and its surroundings in the virtual underwater environment, which were basic parameters required for the application of the surrounding environment estimation. The overall structure of the tactile monitoring system is shown in Figs. 1F and 4D. When the U3DTT first collide with an obstacle in Fig. 6C, a large pulse signal is generated from the CP-TENG sensor at *R*_4_ position in Fig. S16. Then, the impact force was redistributed to the tension network, generating the corresponding signal of the RS-TENG sensors in Fig. S17. Figure 6D and E shows the different positions of collision in real-time sensing (also see Movie S6). As shown in Fig. 6F, the full-cycle output signal was analyzed by the trained DL model to predict the deformation of the U3DTT with the RMSE of 0.76 and then obtain the information of surrounding environment of the underwater vehicle. Note that compared with rod length, the error was acceptable for the tactile system. In addition, these information can be moved to the digital twin for visualizing the deformation of the U3DTT. Furthermore, the U3DTT was used for 3D environment recognition in a narrow tunnel in Movie S7. Figures S18 to S29 show that the predicted data (blue point) track the actual data (red line) well with the RMSE of 0.84, with the corresponding signals of the RS-TENG sensors and the CP-TENG sensors in Figs. S30 and S31. More importantly, the environment-sensing tensegrity device has no influence of optical or sonic issues in the narrow space, thus can be used in dark environments with uneven surfaces and materials with different refractive indices. Overall, these capabilities show the great potential of the underwater tactile perception system based on the U3DTT in autonomously explore an unknown underwater environment.

## Discussion

We have developed the U3DTT based on TENGs and DL-based data analytics to explore an unknown environment, providing a technologically mediated method for the interactions between underwater vehicles and their surroundings, especially when optical or acoustic reflection problems occur in a narrow space. Compared with other 2D tactile devices, our DL-assisted U3DTT provided a variety of attractive characteristics, including multiple DOFs, a high sensitivity, a low cost, fast response times, and conformability.

The U3DTT is a contact-electrification-based device in which the contact area of 2 different materials having opposite triboelectric polarization is periodically changed, which can effectively convert minor tensile forces or pressures into electricity. In particular, the RS-TENG sensor with wire-coiled electrodes was capable of sensing the magnitudes of stretching forces and the position of the force through the device array, showing an excellent sensitivity of 1.8 V/N in a wide strain range of up to 70%. The integration of the wire-coiled electrodes had a high recovery capacity, resulting in good sensing repeatability. In addition, the CP-TENG sensor could measure the pressing motion resulting from a collision and was also resistant to mechanical fatigue. Because of the mechanical design of the thicker flexible cover, it maintained high stability against environmental disturbances. The real-time signal processing capability of the sensory system indicated the potential of achieving interactions between an underwater vehicle and its surroundings. With the aid of the DL to deal with multichannel inputs, the AUV with the U3DTT achieved successful perception and had the average RMSE of about 1.732 for testing data, while the average RMSE for the training data was around 0.3373. In addition, it had an antidisturbance ability to ensure stable measurement. The 3D tactile perception systems based on DL-assisted U3DTT can provide 3D sensing feedback for controlling an underwater vehicle in various tasks, suggesting their potential applicability in vehicles with tactile feedback.

## Methods

### Fabrication of RS-TENG sensor

As shown in Fig. S32, 100 ml of part A and 100 ml of part B silicone rubber were mixed in a Petri dish. Then, a vacuum pump was used to vacuum the mixture to 0.1 MPa for 3 min. When the evacuation of the mixture was complete, the mixture was poured into 2 half-circular molds manufactured by 3D printing. A commercial wire coil (Ag-plated carbon steel) was inserted into the mixture. After combining these 2 half-circular tubes for encapsulation, the silicone rubber was cured naturally at room temperature. The cured silicone rubber was demolded from the molds. CNTs with a resistivity of 1, 412 μΩ-m were added to silicone rubber and subjected to ultrasonic mixing for 5 min to uniformly disperse the CNTs in the silicone rubber. A wire coil was dipped into the CNT dispersed solution for 1 min, followed by air for curing naturally at room temperature. Finally, a latex tube with a 3-mm wall thickness was used for encapsulation.

### Fabrication of CP-TENG sensor

The flexible cover was made of Dragonskin 10, as shown in Fig. S33. Specifically, 40 ml of part A and 40 ml of part B silicone rubber were mixed in a Petri dish. Then, a vacuum pump was used to vacuum the mixture to 0.1 MPa for 3 min. When the evacuation of the mixture was finished, the mixture was poured into a mold manufactured by 3D printing. The silicone rubber was cured naturally at room temperature. Finally, a commercial checkerboard-shaped Cu block and hemispherical cover were assembled using Kafuter K^-704^ glue.

### Fabrication of the U3DTT

The tensegrity structure was an icosahedron mainly composed of 6 rigid bars, 24 RS-TENG sensors, and 12 CP-TENG sensors. Multiple sensors could not be mounted on the same node unless additional safeguards were used to ensure that they did not interfere with one another. As a result, each end of each bar was equipped with a flange, as shown in Fig. S34. Four studs were required to secure several sensors to each flange. The RS-TENG sensor was placed into the groove of a metal lock and then connected to a stud installed on the flange using a steel wire to guarantee that the RS-TENG sensor remained attached to the flange during recurrent elongation and contraction periods. The CP-TENG sensor was mounted on the flange’s center. It is a location for markers identified by the motion capture system.

### Electric measurement and characterization

Field-emission scanning electron microscopy (OLS4000) is used to characterize the surface morphology of silicone rubber-CNT composite. For the electric output measurement of TENG, a linear motor (LINMOT EI200-P01) equipped with a force sensor (LZ-WL2) is used to simulate a cyclic stretching/pressing force. The voltage signal is measured using a Keithley (6514) electrometer. The NI-6259 is used to gather data. The software platform is built on LabVIEW, which enables the control and analysis of real-time data gathering.

## Data Availability

We declare that the main data supporting the findings of this study are available within the article and Supplementary Materials. Extra data are available from the corresponding authors on reasonable request.

## Supplementary Materials

Supplementary 1 Supplementary Notes Figs. S1 to S34 Table S1
