# Supplementary material for: Deep-Learning-Assisted Underwater 3D Tactile Tensegrity
Source: Research (Wash D C). 2023 Feb 27;6:0062. doi: 10.34133/research.0062 (PMC10013964; doi:10.34133/research.0062)
Supplement: Supplementary 1 — Supplementary Notes Figs. S1 to S34 Table S1 [file research.0062.f1.pdf]

Supplementary Materials

Supplementary Notes

Note S1. Analysing degrees of freedom for the TENG-based tensegrity sensor

Since rotation and translation actions had no effect on the shape of the TENG-based tensegrity sensor, we only considered the relative position and orientation of n rods with respect to the AUV. Note that all cables and rods were geometrically equivalent, and comprise an icosahedron which were grouped into two categories on the surface: eight closed equilateral triangles and 12 open isosceles triangles. The total DOF is determined to be $5n$, without taking into account the rotation of the rods around their axes. However, point-symmetric shapes rotated around the reference AUV were identical, the DOF of the TENG-based tensegrity sensor can be finally given

$$DOF = 5n - 1. \tag{2}$$

The DOF was derived as 29, guaranteeing widespread use in the interaction between the underwater vehicle and the environment.

Supplementary Figures

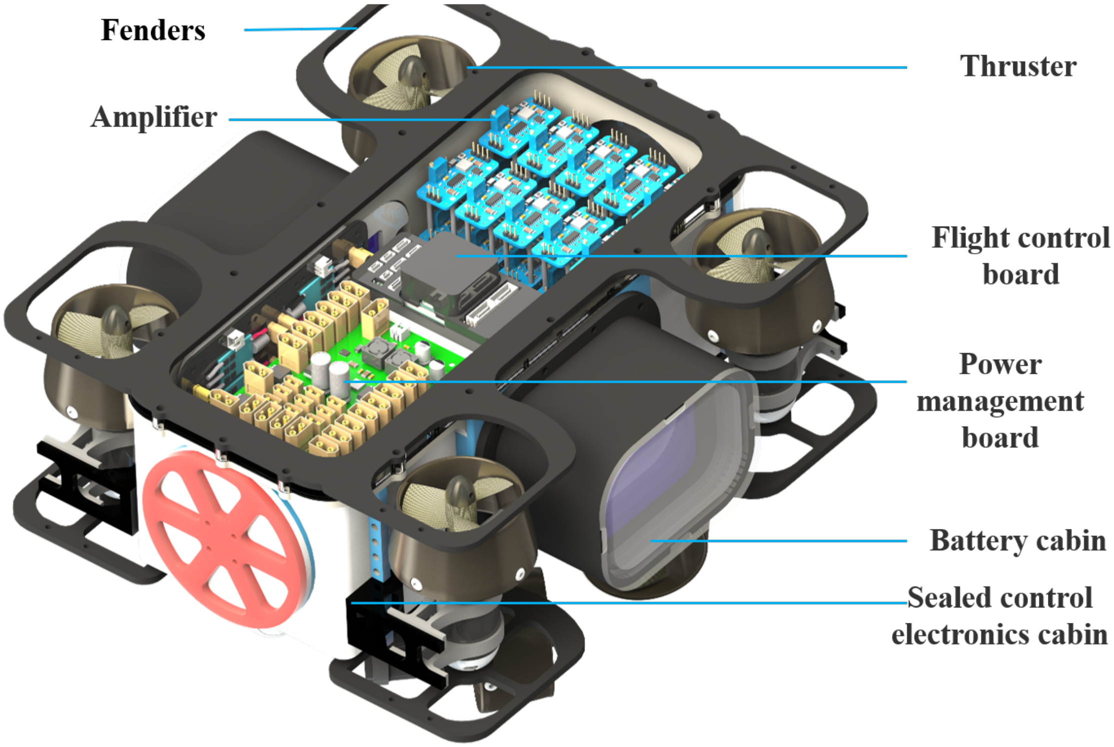

Figure S 1: Structural diagram of AUV.

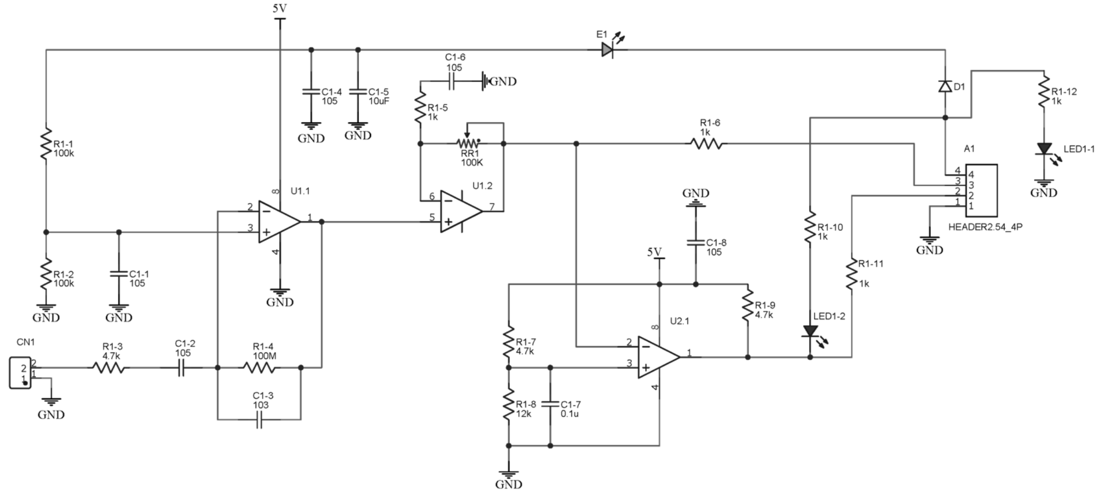

Figure S 2: The signal-conditioning circuit.

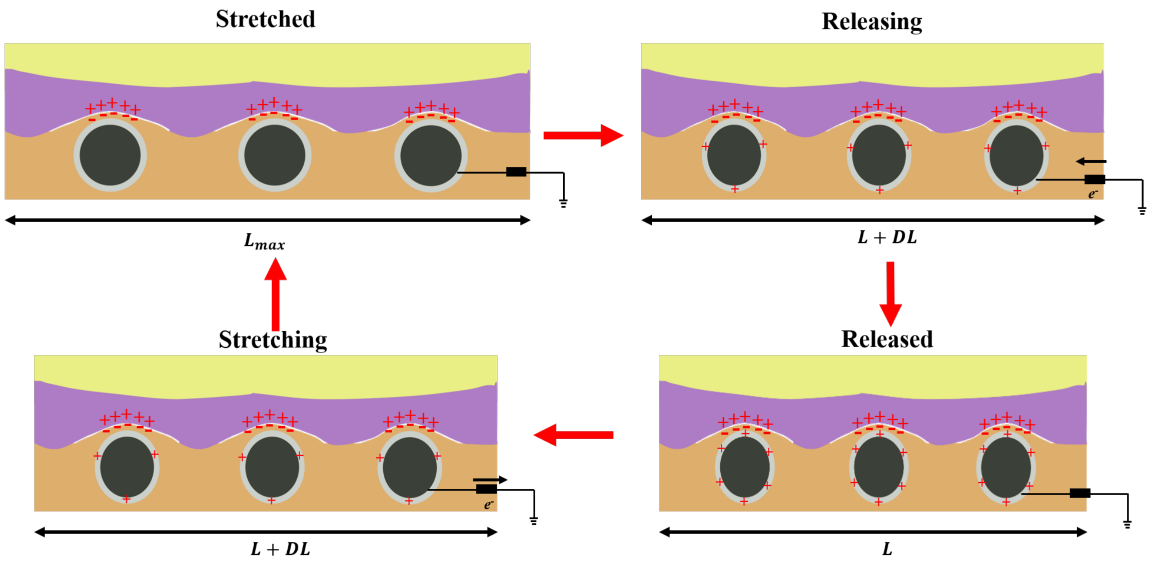

Figure S 3: Working mechanism for generating electricity under short-circuit conditions.

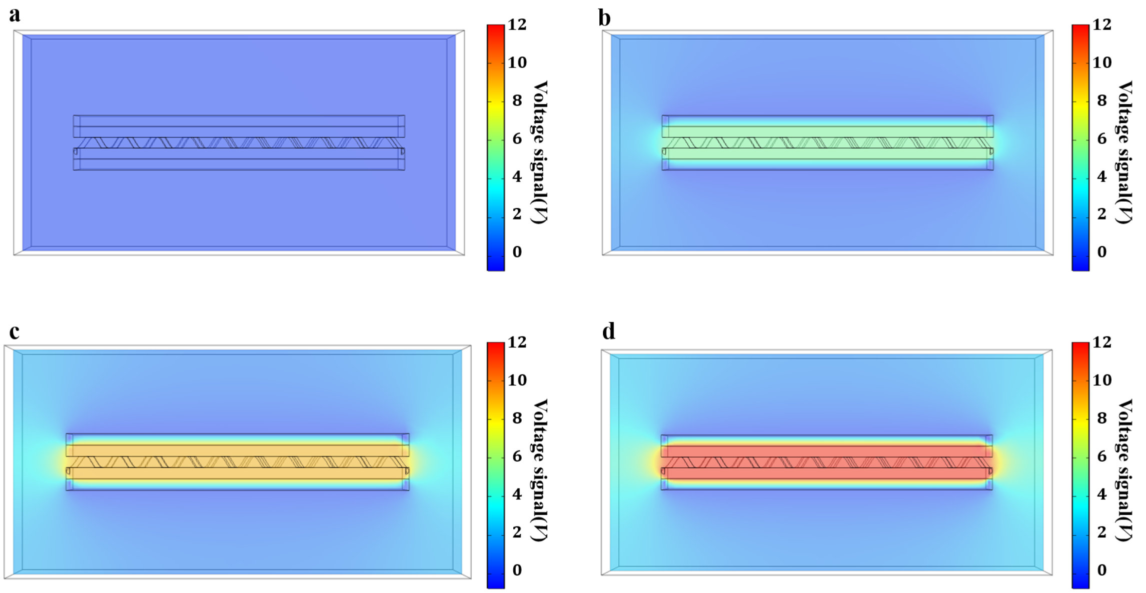

Figure S 4: Electric potential distributions for the stretching RS-TENG under different strains. a, Strain = 0%. b, Strain = 30%. c, Strain = 50%. d, Strain = 70%.

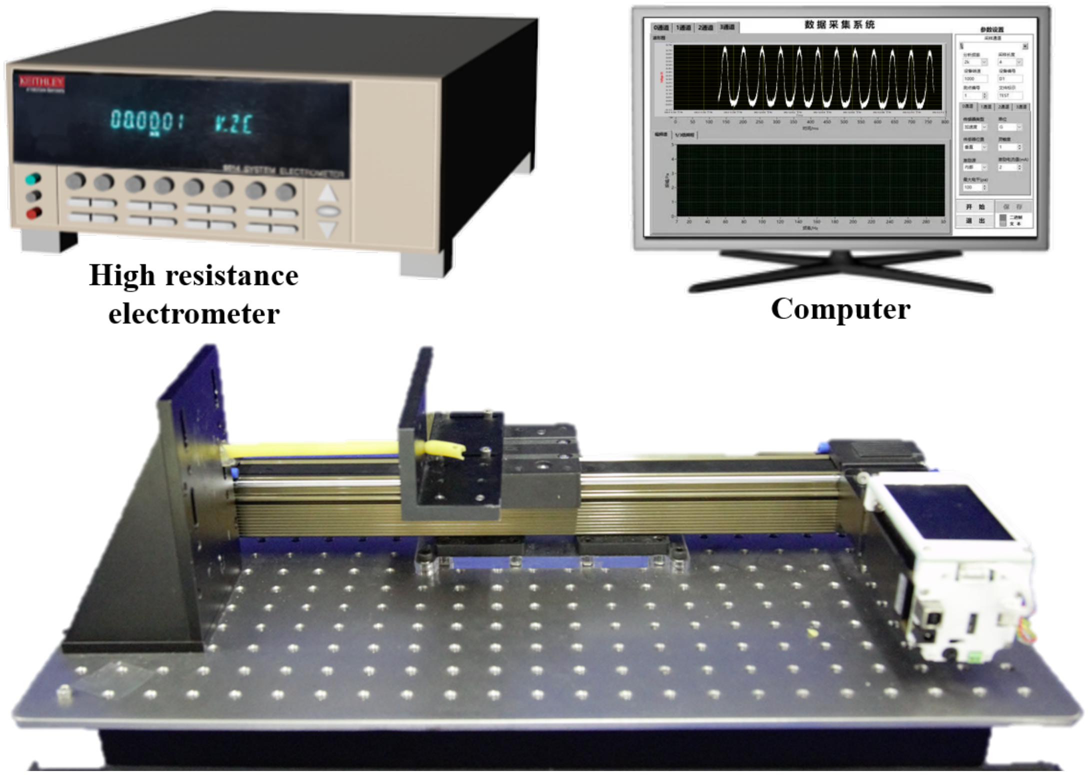

Figure S 5: Schematic of the experimental setup.

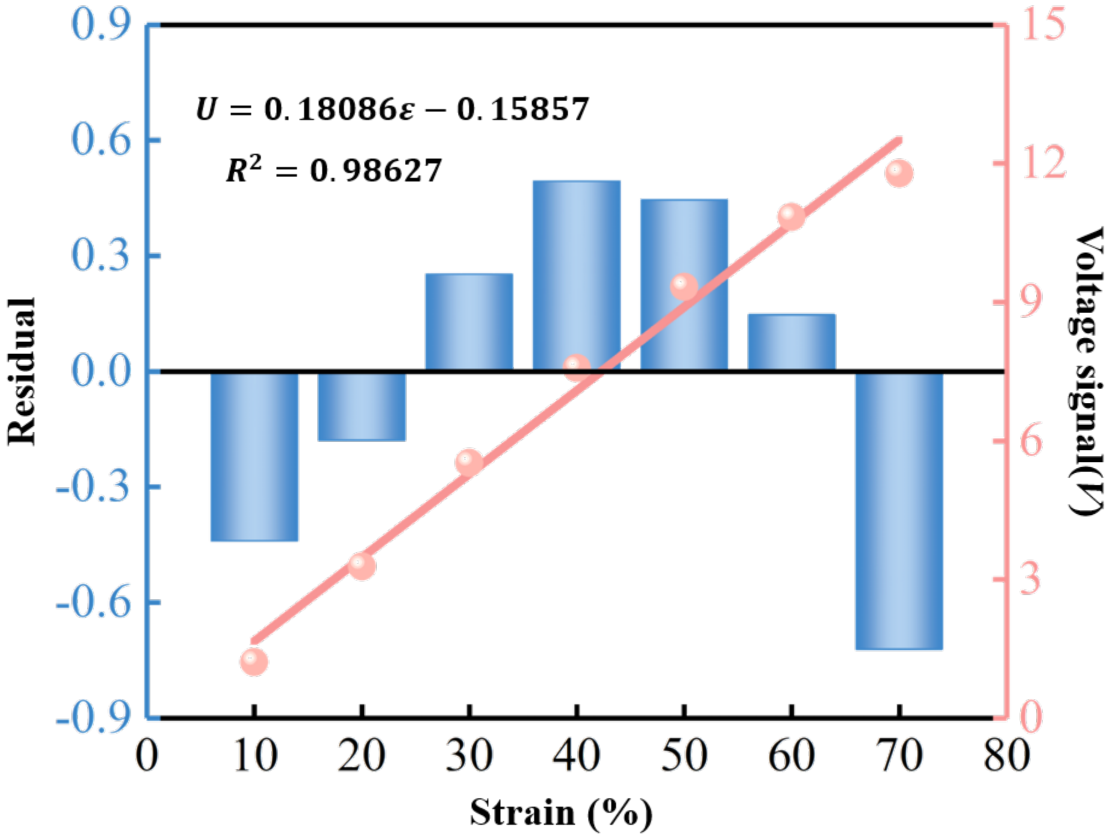

Figure S 6: Fitted linear relationship between voltage signal and strain.

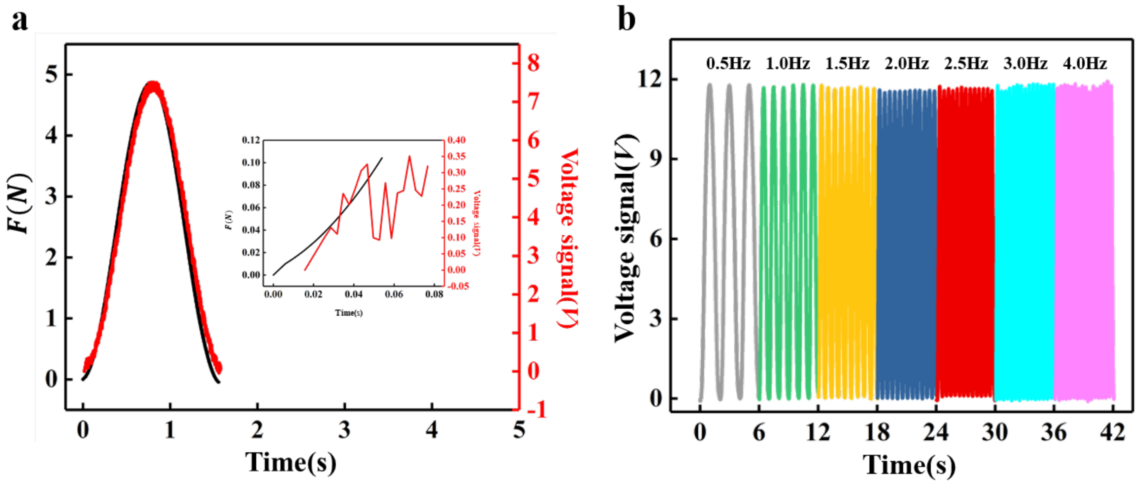

Figure S 7: (a) Response time measurement of the RS-TENG sensor. (b) Output voltage signals of the stretching RS-TENG sensor with varying frequencies at a constant strain of 70%.

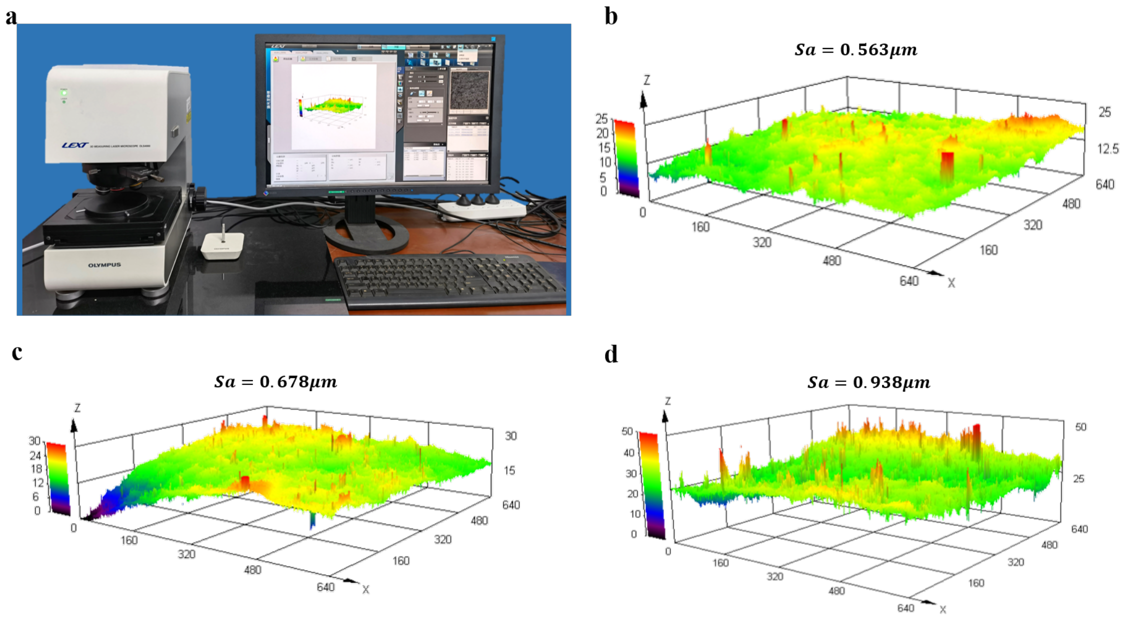

Figure S 8: Cross-sectional SEM image of silicone rubber-CNTs composite. a, Hardware setup. b, c, d, Different CNTs concentration (b) 0.005g/ml, (c) 0.01g/ml, (d) 0.015g/ml.

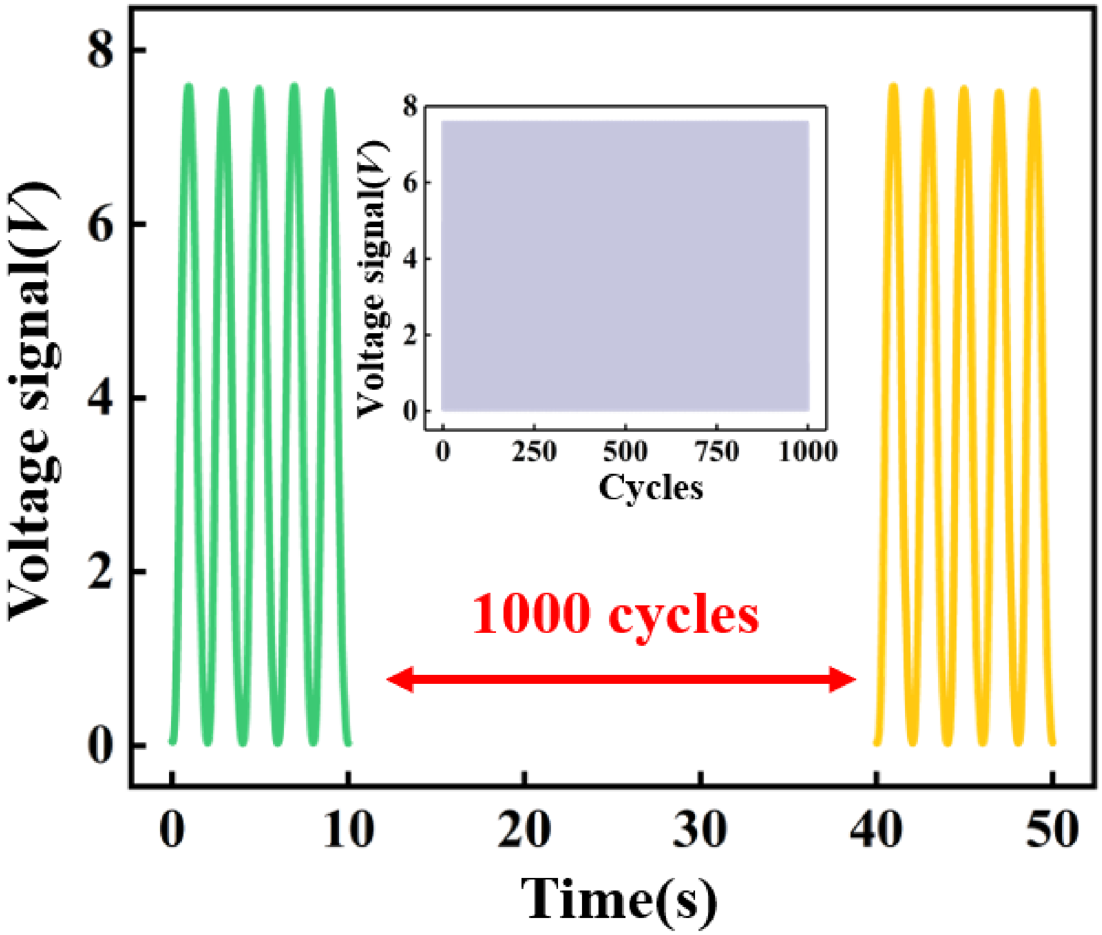

Figure S 9: Mechanical durability test for up to 1000 continuous stretch–release cycles.

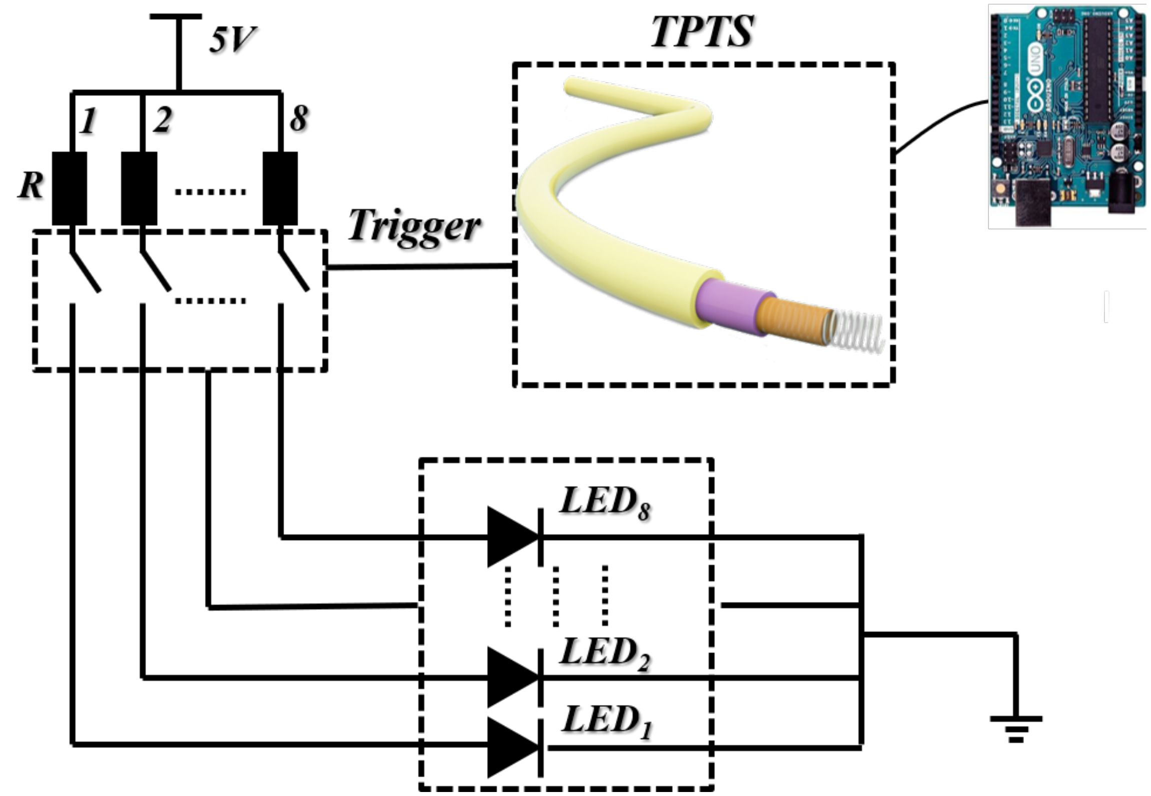

Figure S 10: Electronic module used for potential application demonstrations.

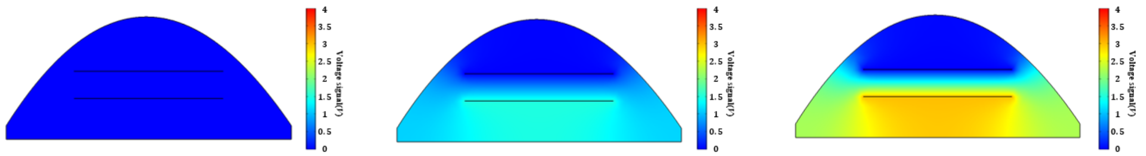

Figure S 11: Electric potential distributions for the CP-TENG under different pressing force. a, Force = 0 N. b, Force = 1 N. c, Force = 17 N.

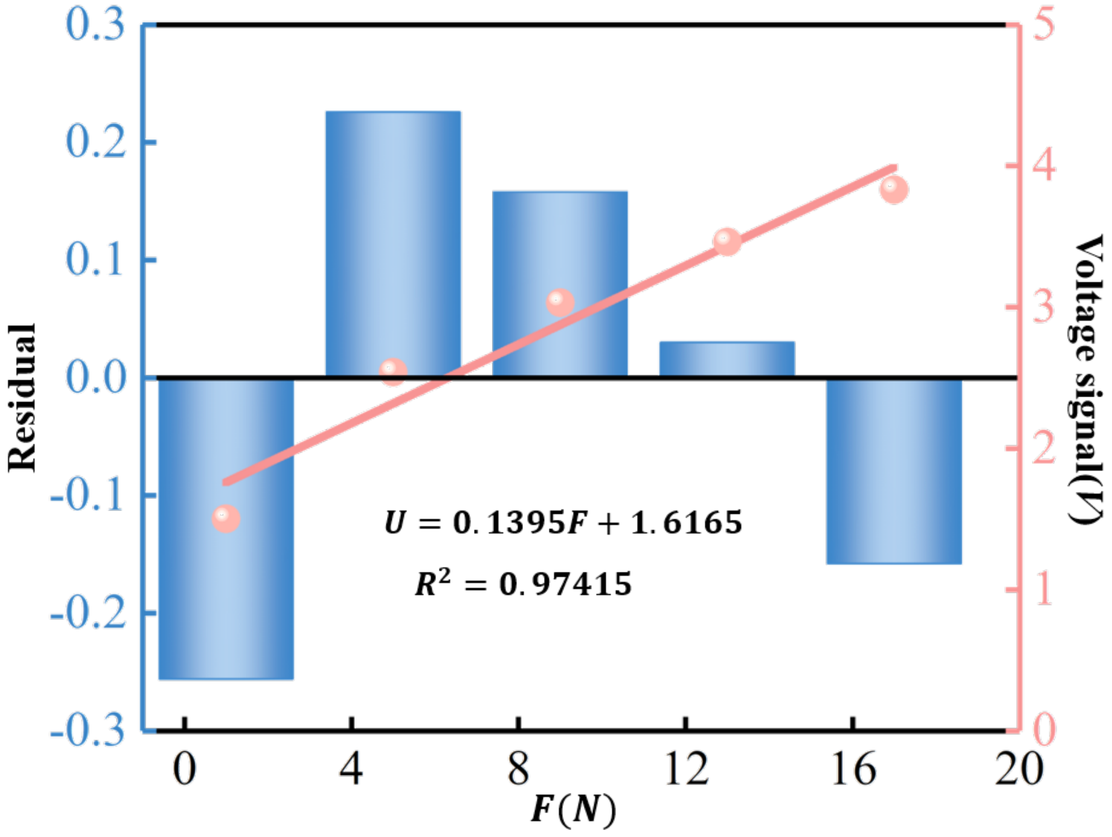

Figure S 12: Fitted linear relationship between voltage signal and pressing force.

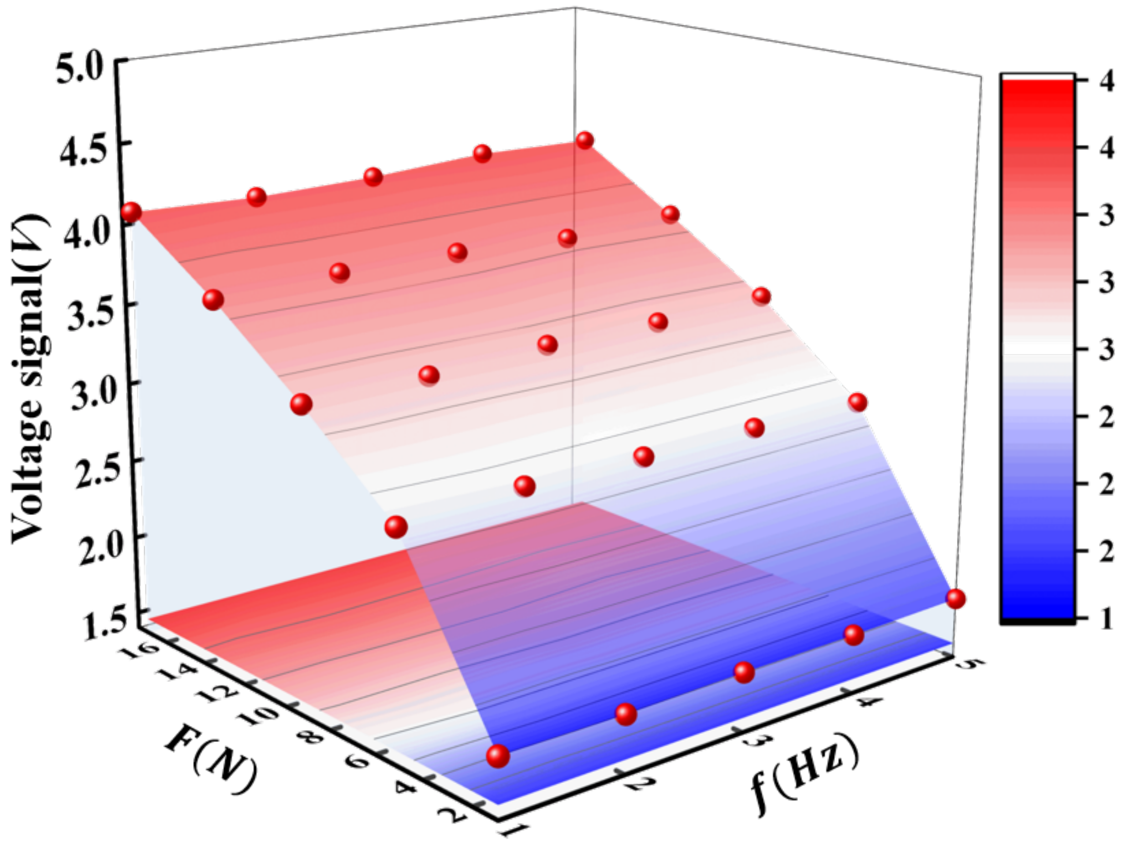

Figure S 13: Relationship between the output signal, pressing force and pressing frequencies.

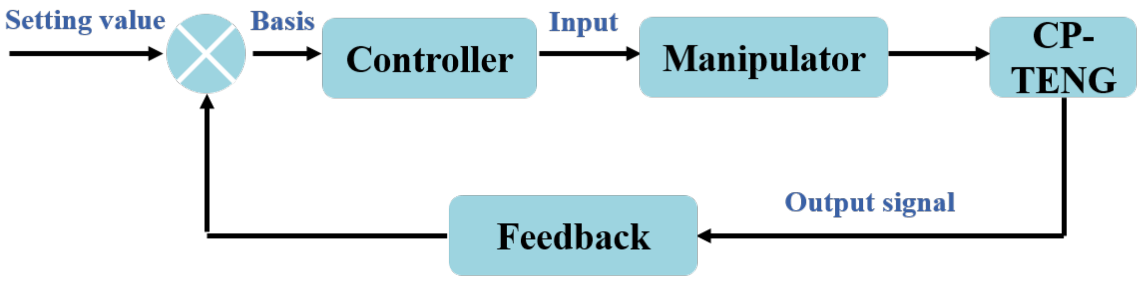

Figure S 14: The closed-loop control system for the manipulator.

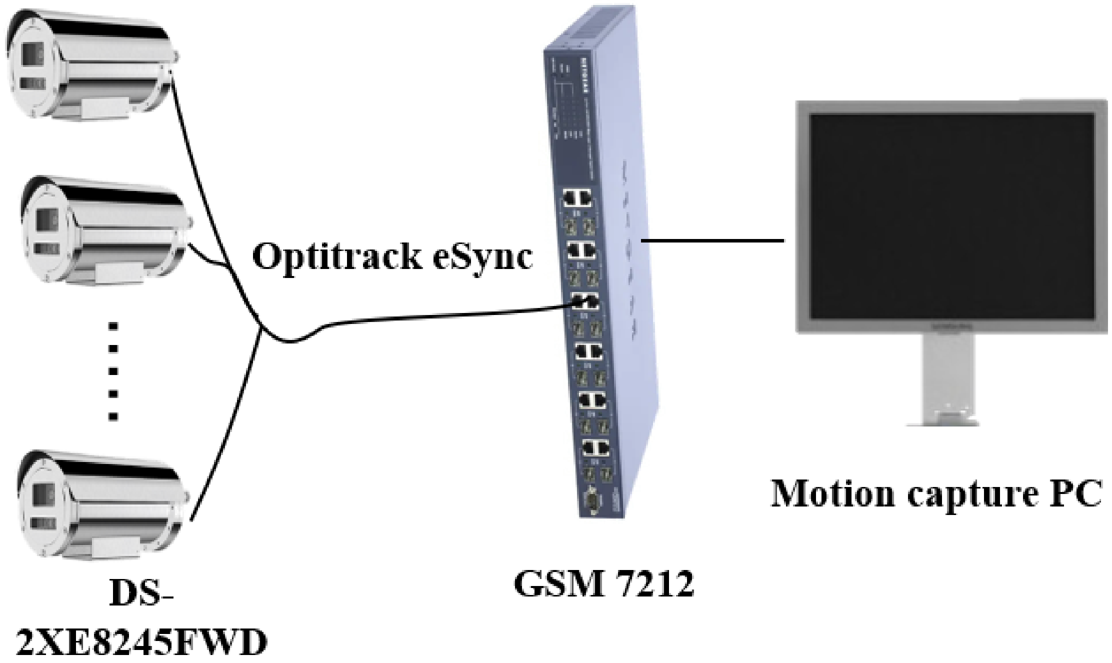

Figure S 15: Hardware architecture of motion capture system.

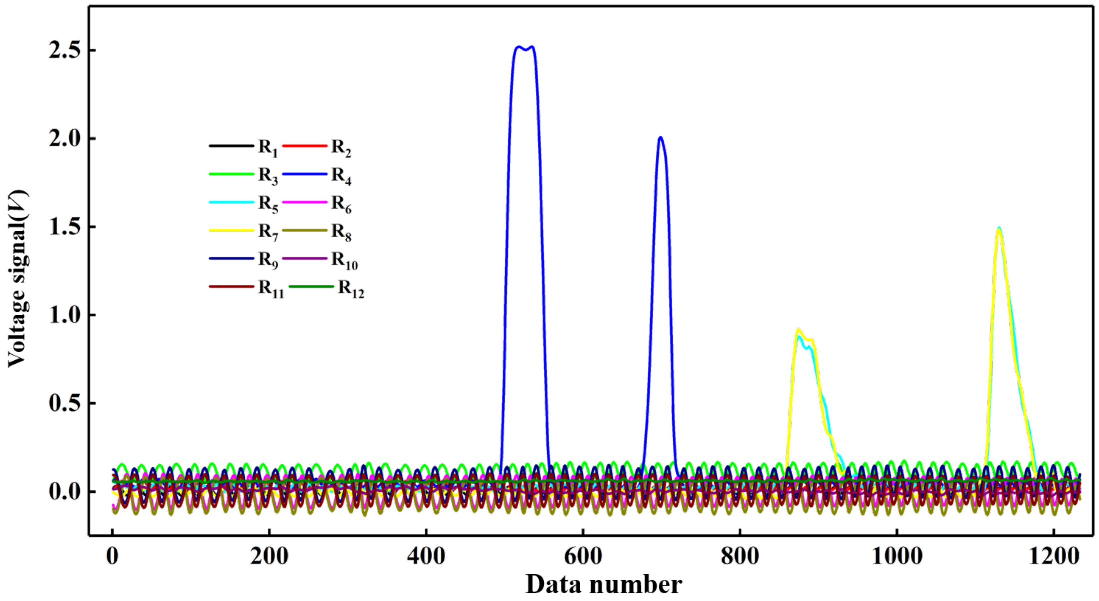

Figure S 16: Output voltage of the CP-TENG sensors in the case of collisions.

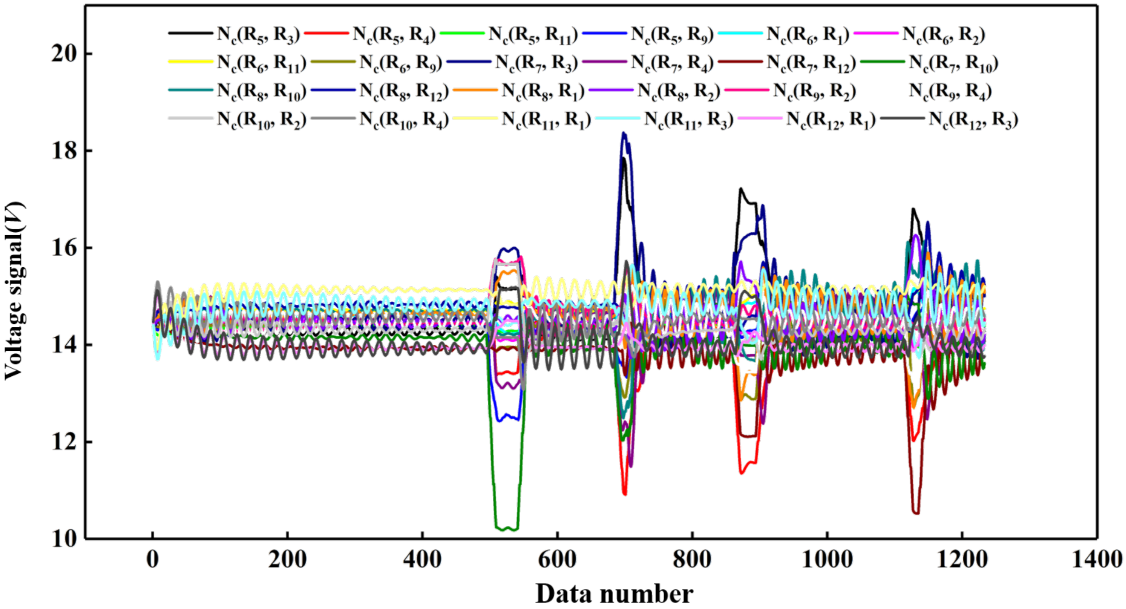

Figure S 17: Output voltage of the RS-TENG sensors in the case of collisions.

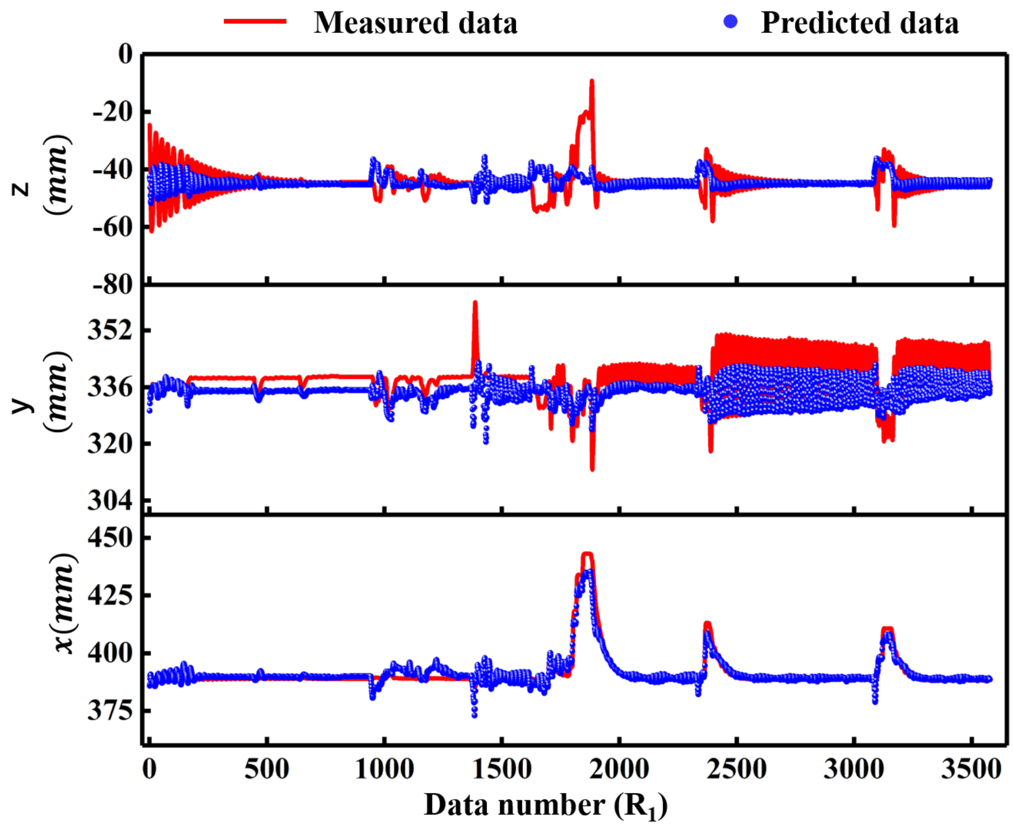

Figure S 18: Position predictions for R_1.

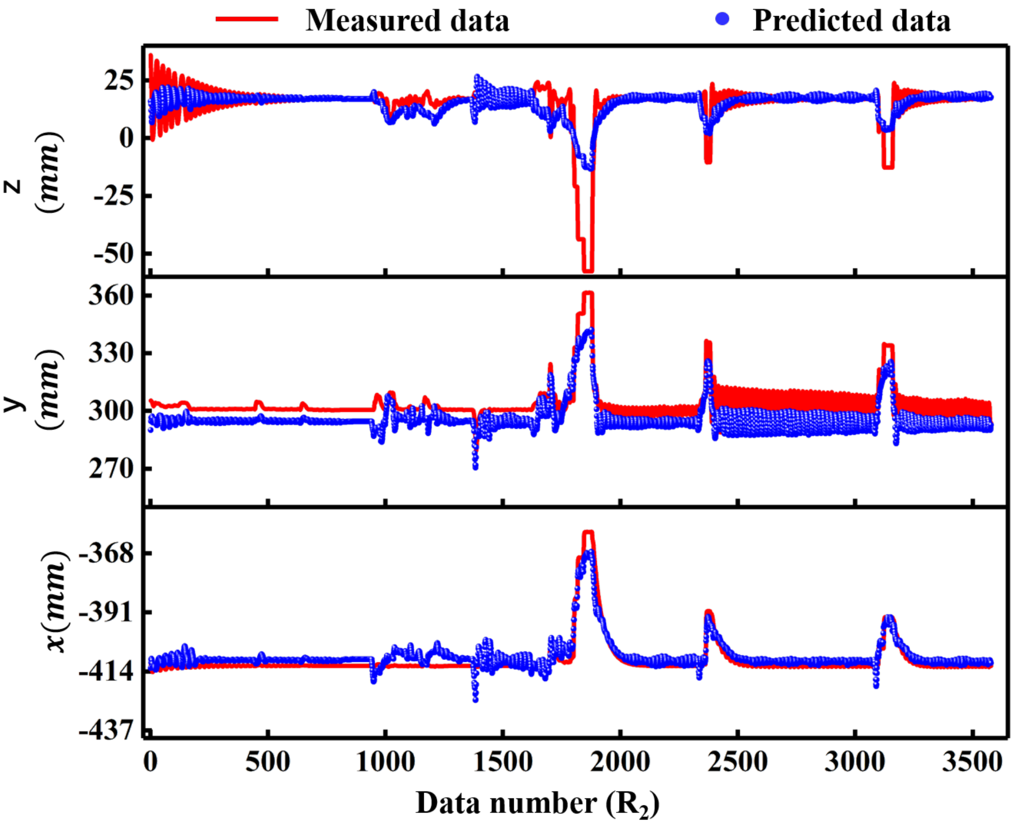

Figure S 19: Position predictions for R_2.

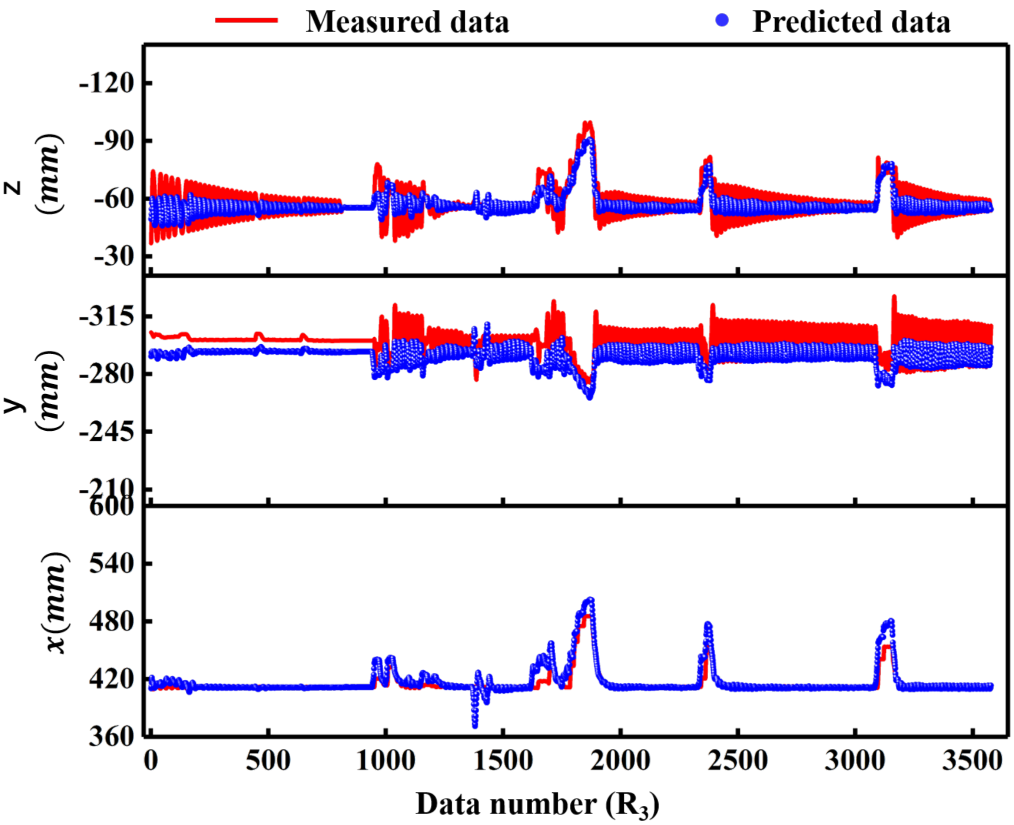

Figure S 20: Position predictions for R_3.

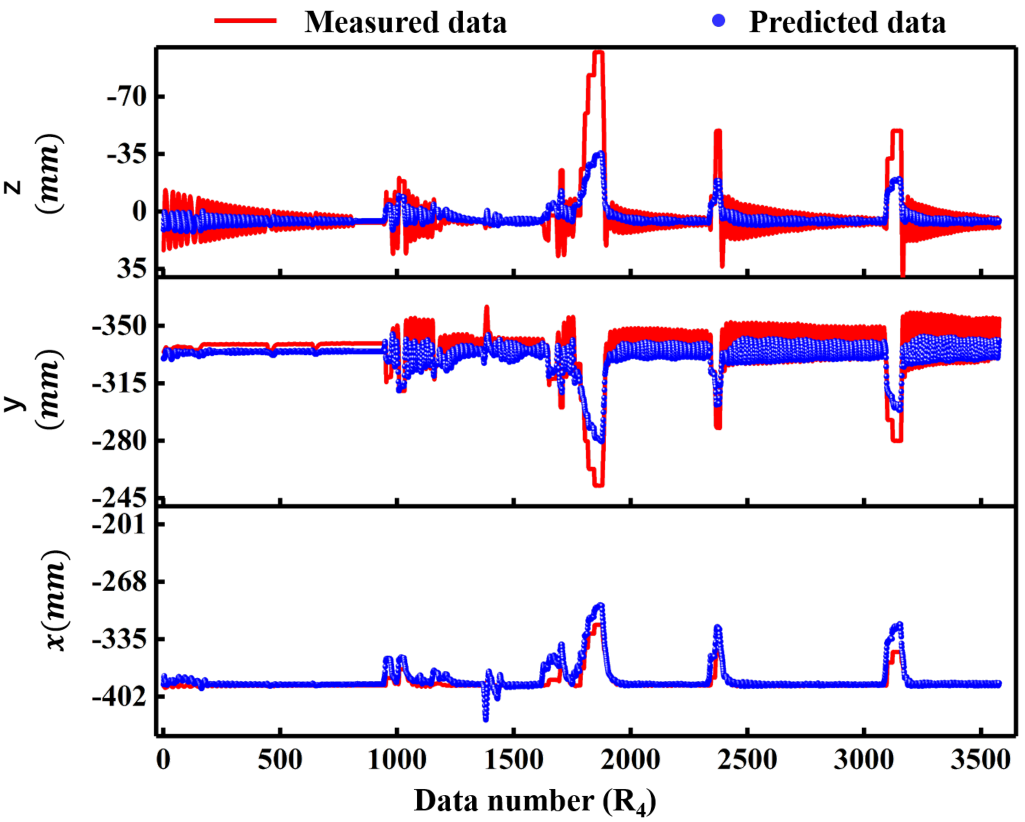

Figure S 21: Position predictions for R_4.

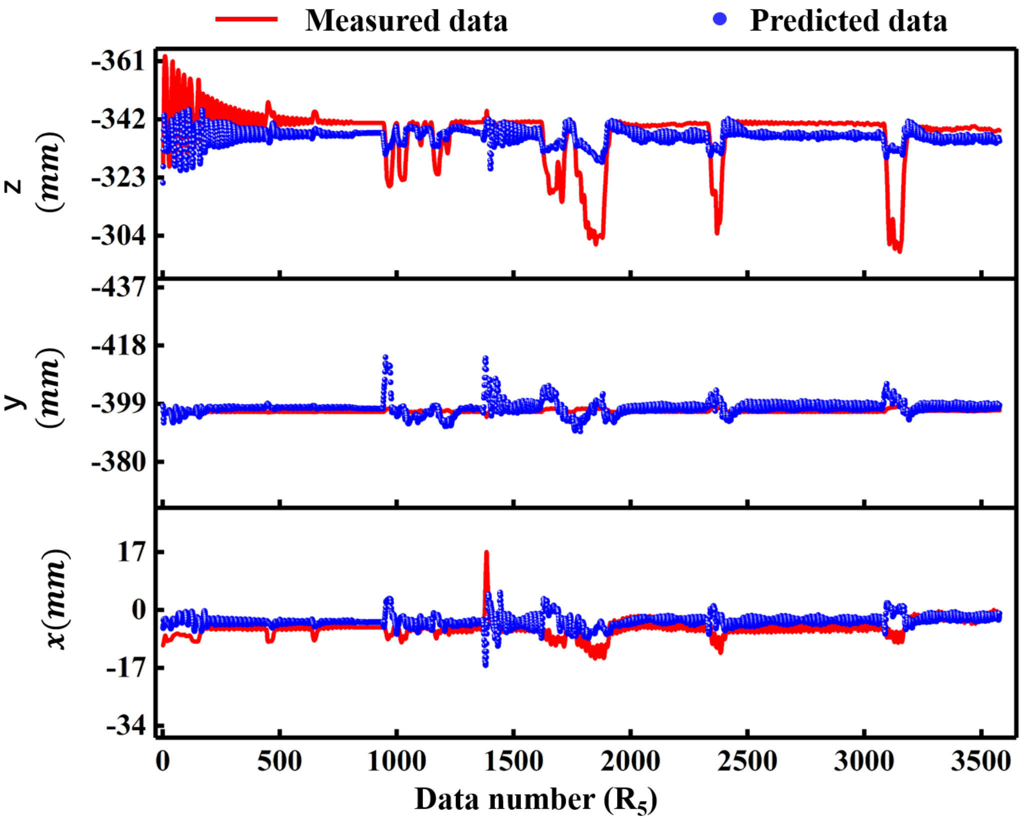

Figure S 22: Position predictions for R_5.

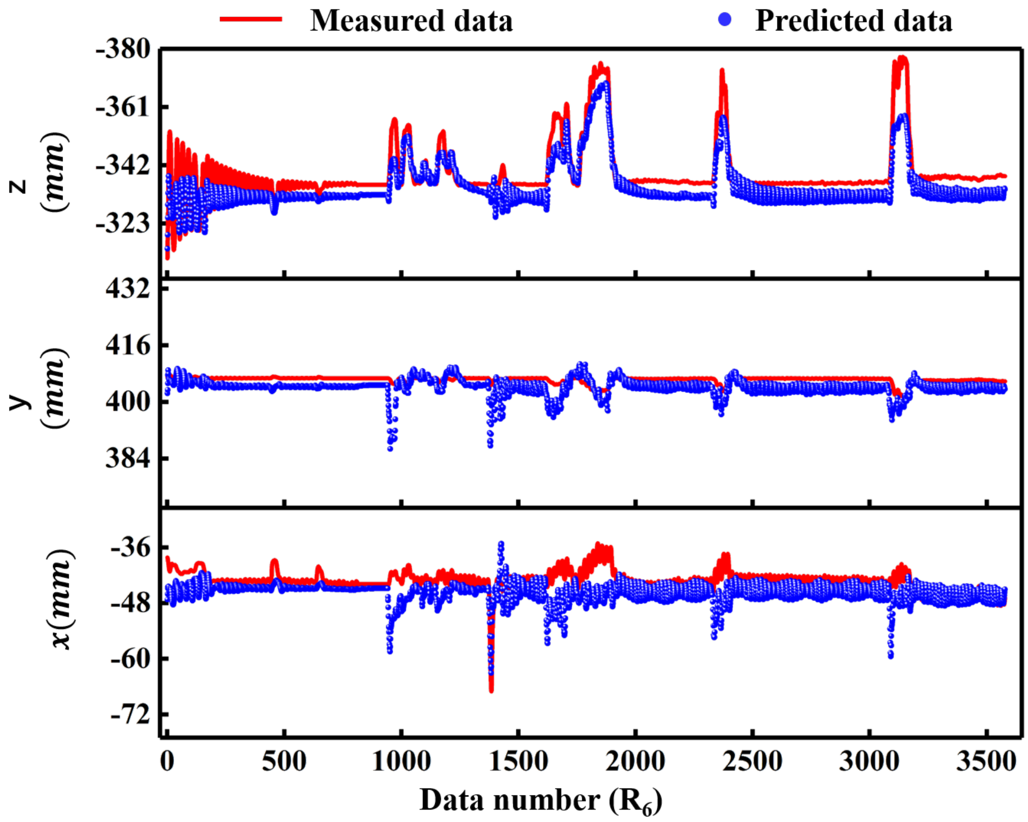

Figure S 23: Position predictions for R_6.

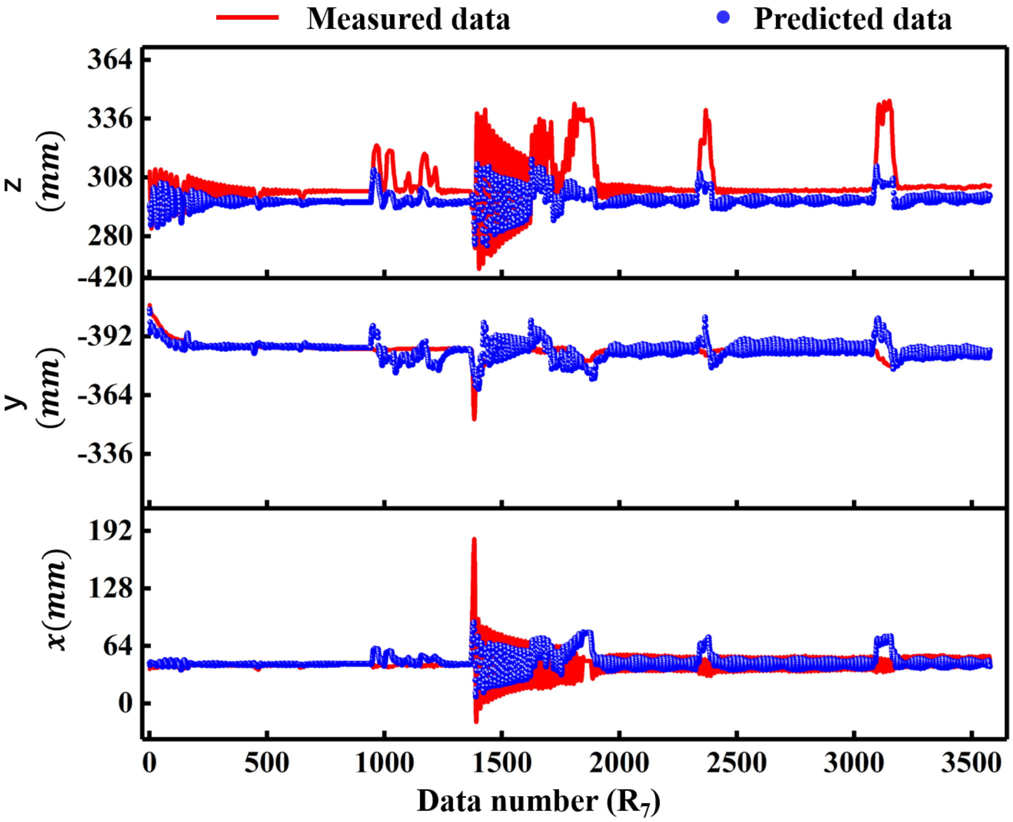

Figure S 24: Position predictions for R_7.

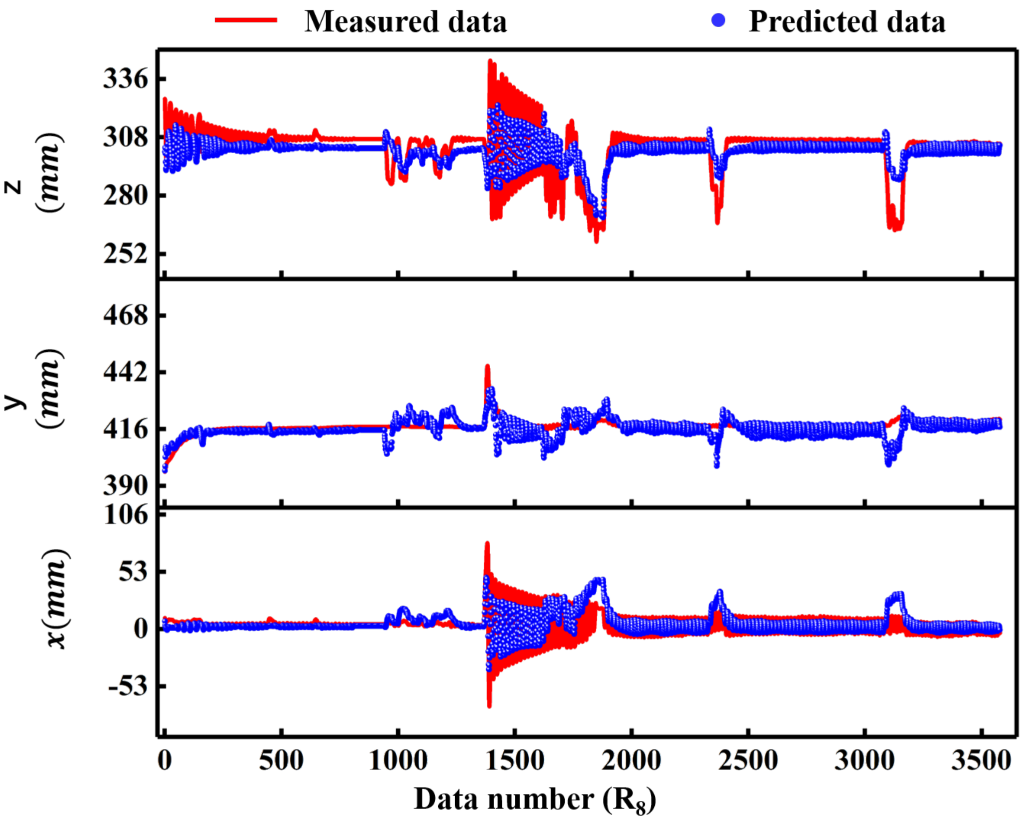

Figure S 25: Position predictions for R_8.

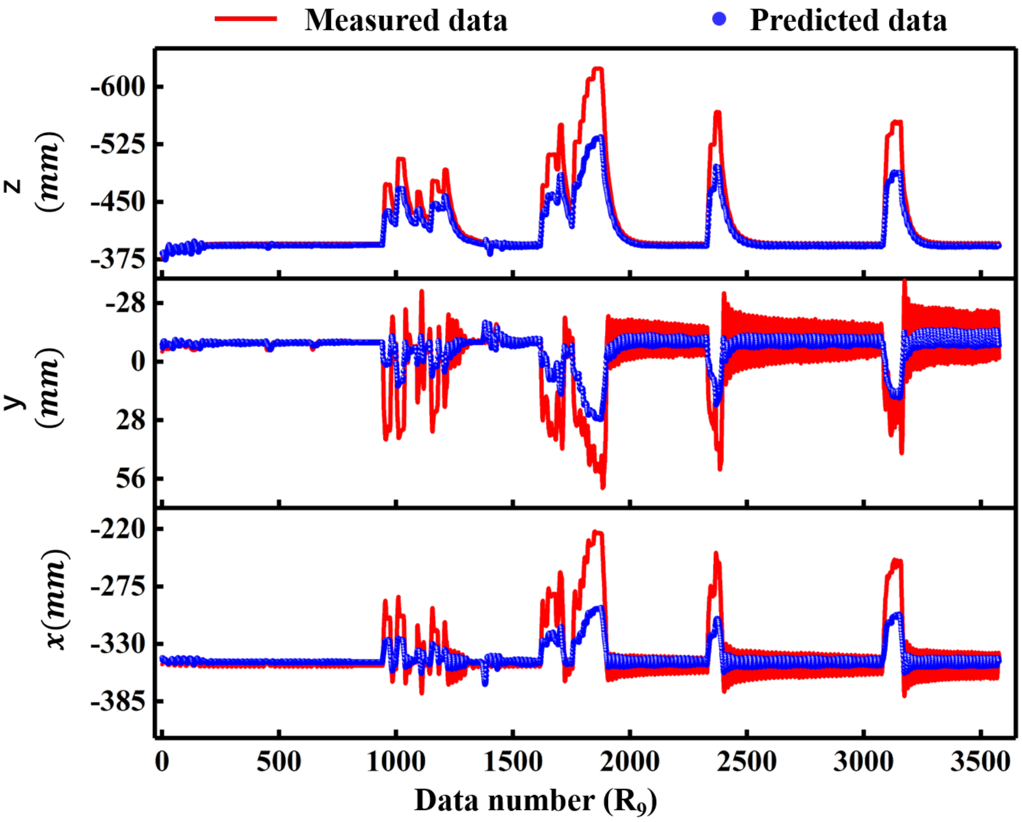

Figure S 26: Position predictions for R_9.

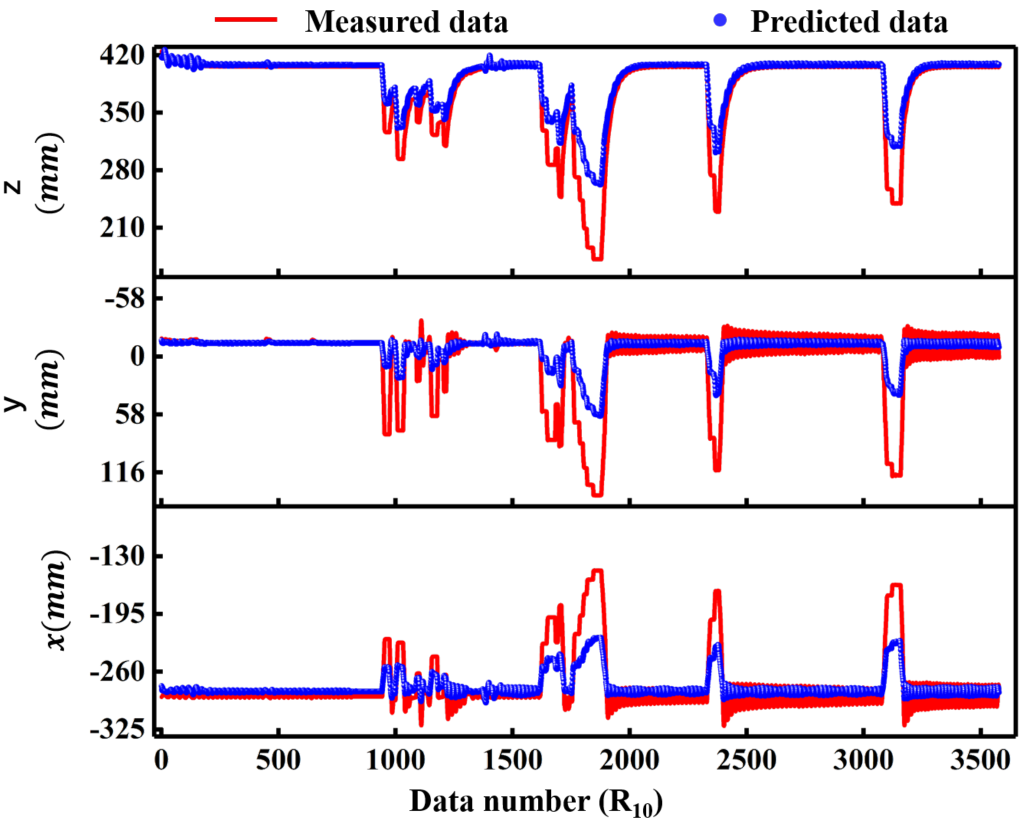

Figure S 27: Position predictions for R_{10}.

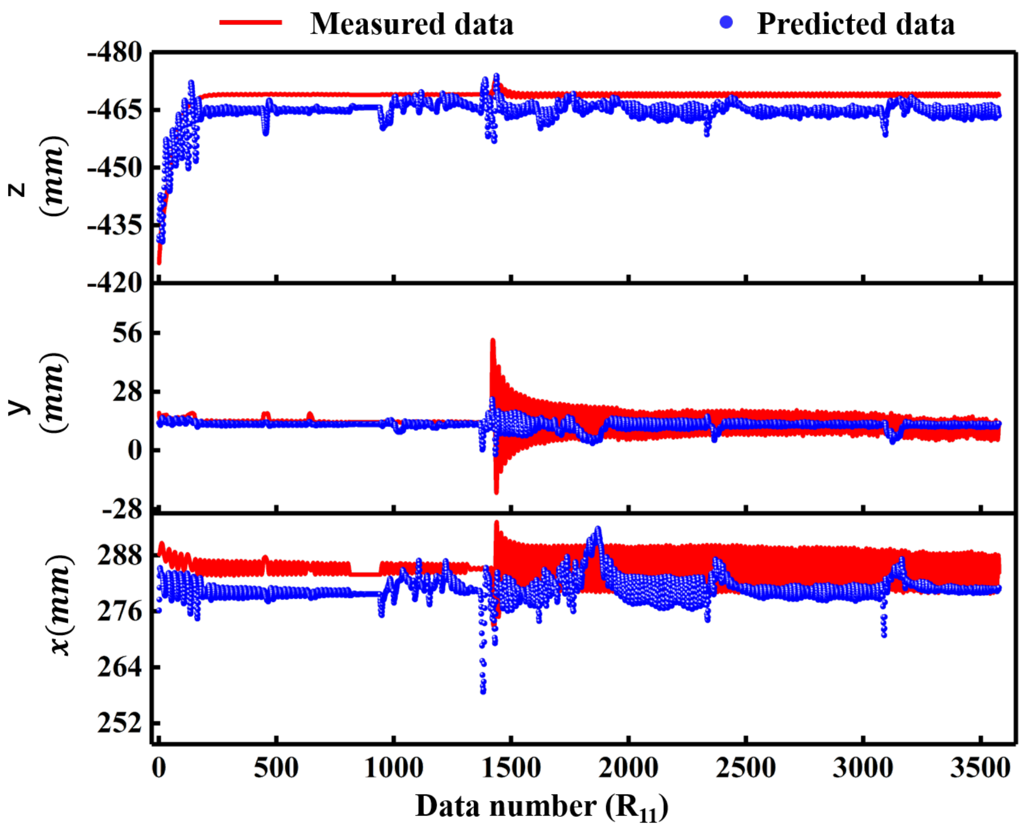

Figure S 28: Position predictions for R_{11}.

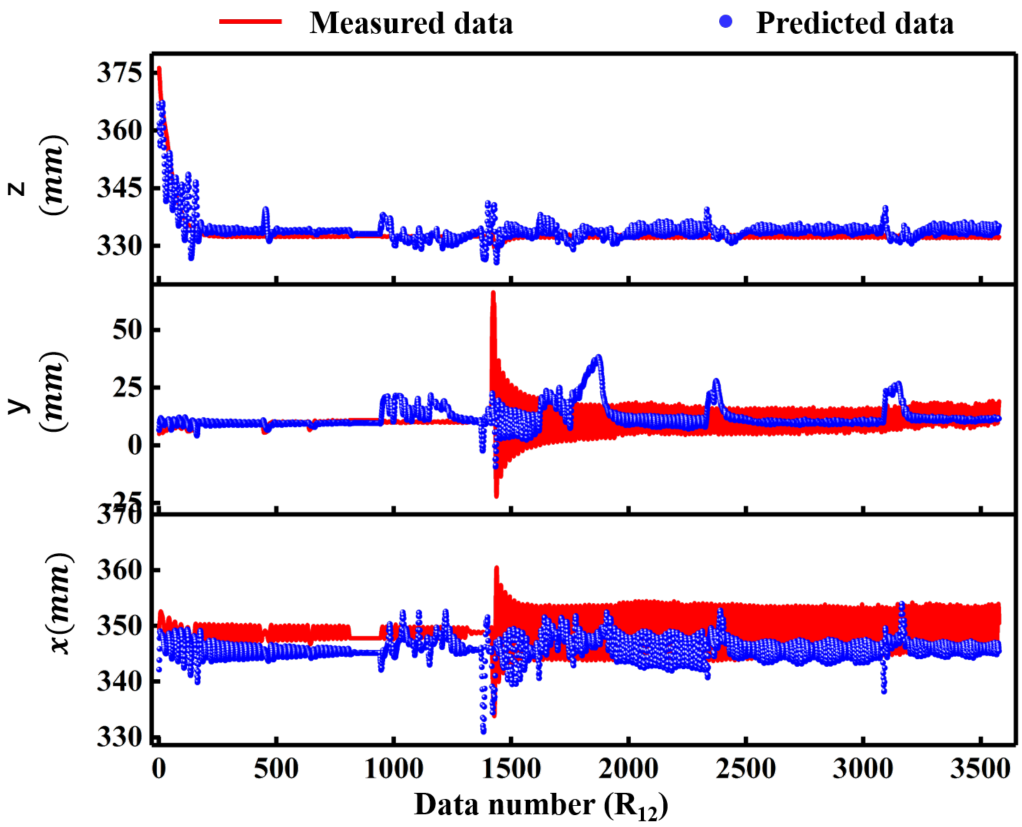

Figure S 29: Position predictions for R_{12}.

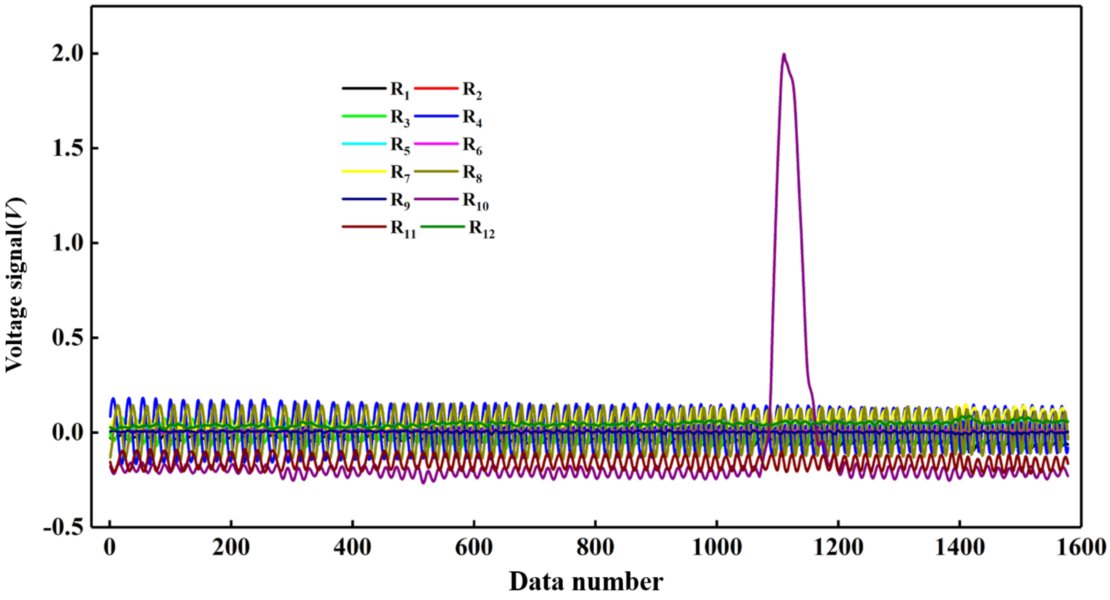

Figure S 30: Output voltage of the CP-TENG sensors in the case of collisions.

Supplementary Tables

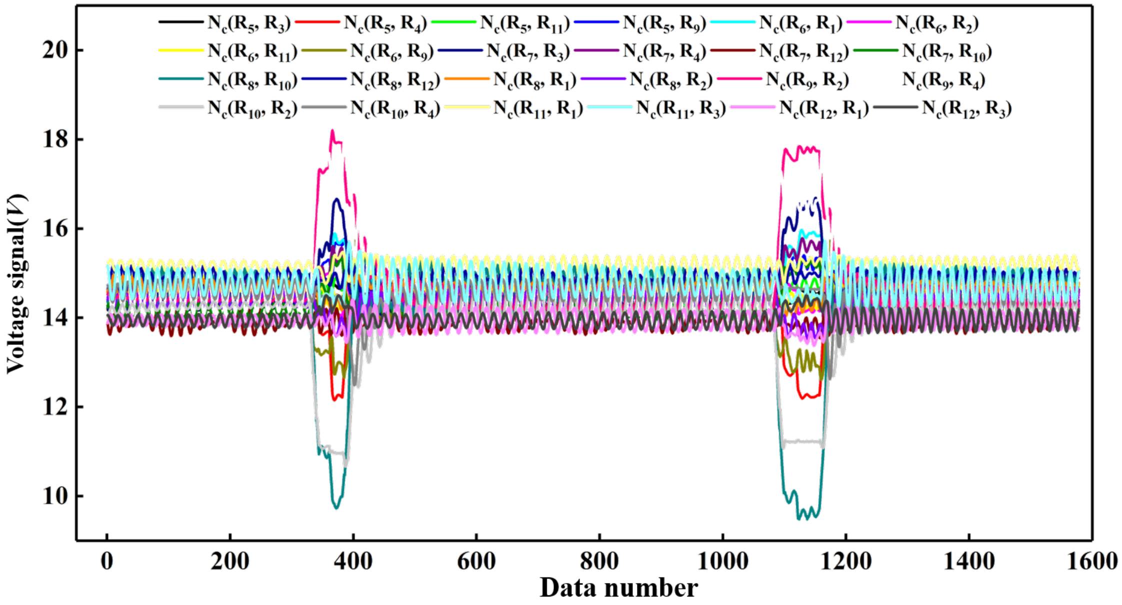

Figure S 31: Output voltage of the RS-TENG sensors in the case of collisions.

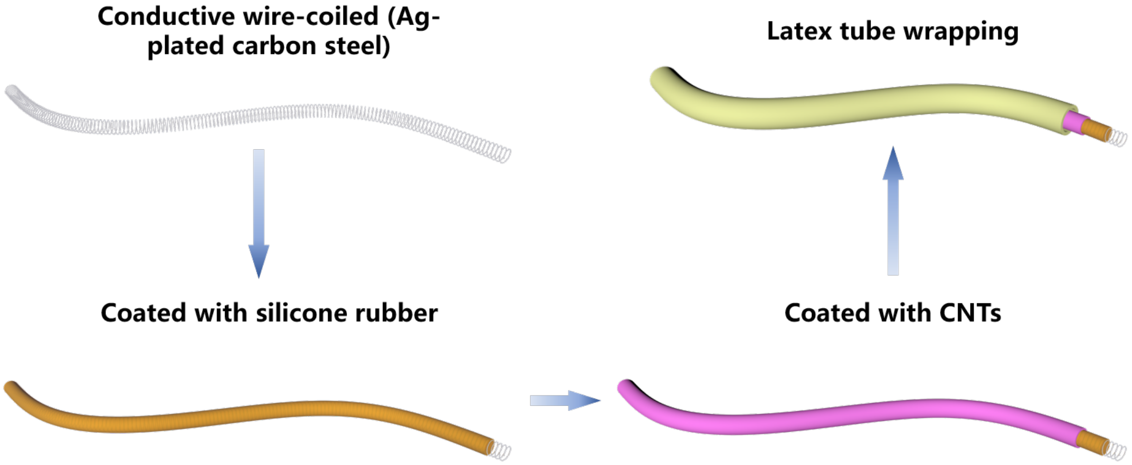

Figure S 32: RS-TENG fabrication and assembly.

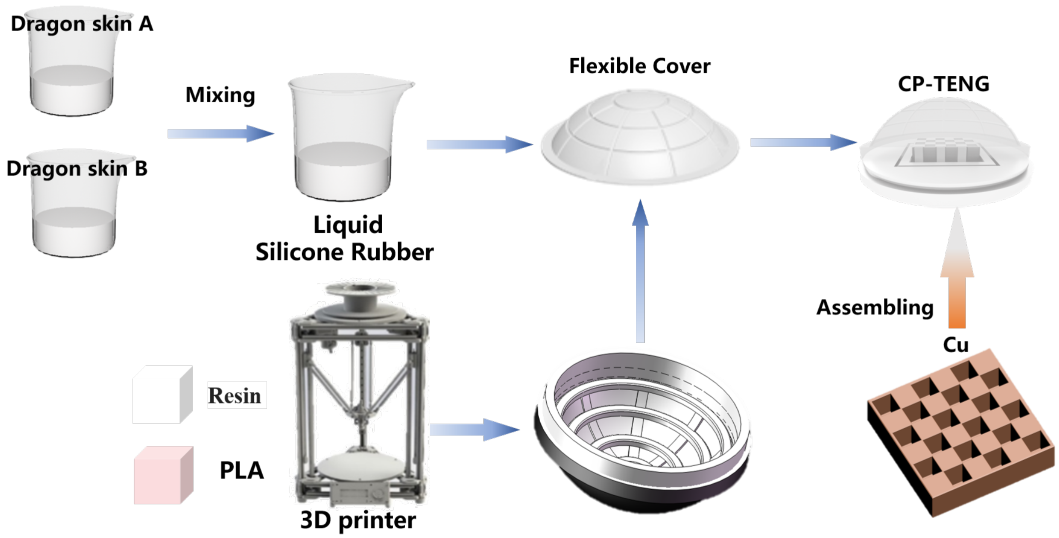

Figure S 33: CP-TENG fabrication and assembly.

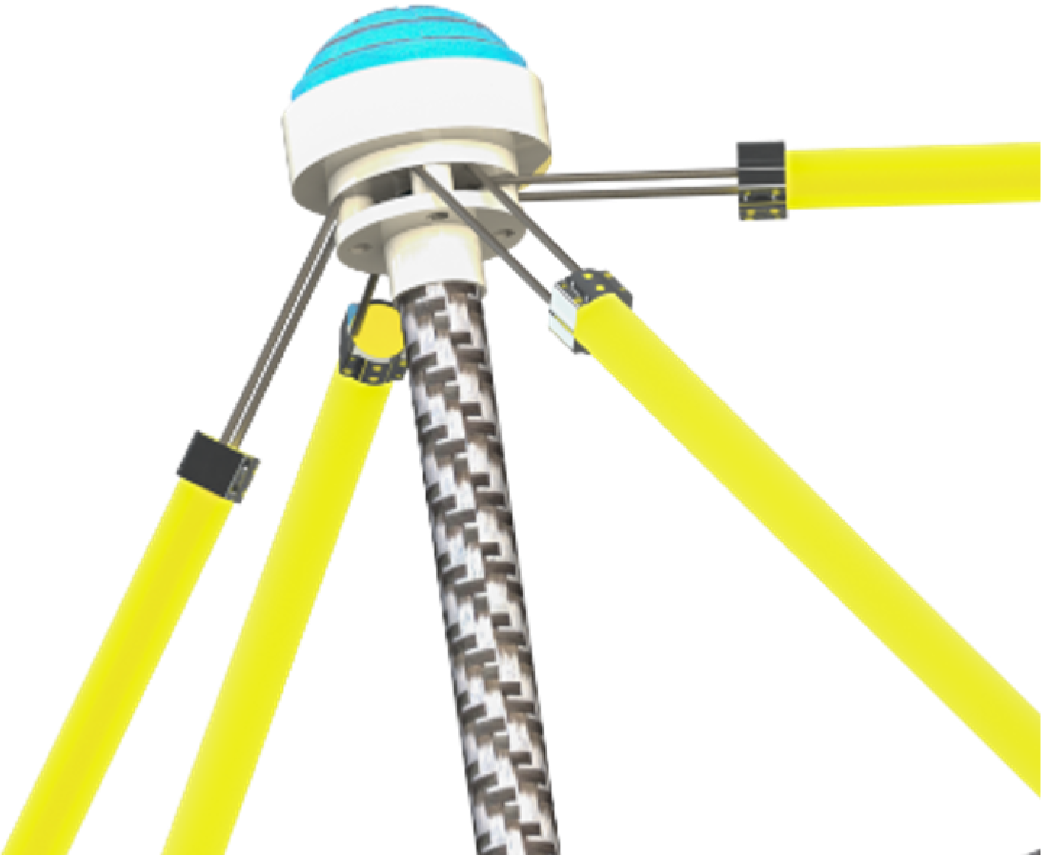

Figure S 34: structural end of each bar.

Supplementary Table 1: Root-mean-square error for node position

	Case number	RMSE
Means of training data	——	0.3373
Means of testing data	——	1.732
Worst of testing data	Case 41	3.7
Best of testing data	Case 47	0.9
